# Defective autophagy in GNE myopathy is rescued by inhibition of noncanonical Akt–mTORC1 activation across multiple isogenic models

**DOI:** 10.1038/s12276-026-01701-7

**Published:** 2026-04-10

**Authors:** Dong-Woo Kim, Eun-Ji Kwon, Hyuk Kwon, Jumee Kim, Hyuk-Jin Cha

**Affiliations:** 1 College of Pharmacy, Seoul National University, Seoul, Republic of Korea; 2 College of Pharmacy, Sookmyung Women’s University, Seoul, Republic of Korea; 3 Research Institute of Pharmaceutical Sciences, Seoul National University, Seoul, Republic of Korea

**Keywords:** Experimental models of disease, Stem-cell research, Translational research, Drug development, Stem-cell biotechnology

## Abstract

GNE myopathy is a recessive autosomal disease caused by mutations in glucosamine (UDP-*N*-acetyl)-2-epimerase/*N*-acetylmannosamine kinase (GNE), characterized by impaired sialic acid biosynthesis and the formation of rimmed vacuoles. Similar to other autophagic vacuolar myopathies, defective autophagy has been implicated in disease pathogenesis; however, the underlying molecular mechanisms remain poorly understood. By performing transcriptome analysis on two independent GNE myoblast models derived from human pluripotent stem cells, we identified multiple autophagy-related gene sets as pathogenic signatures of GNE myopathy. These predictions were biochemically validated using *Gne*-knockout C2C12 myoblasts. Mechanistically, our data reveal that aberrant activation of the noncanonical AKT–mTORC1 pathway—driven by excessive extracellular matrix production—induces inhibitory phosphorylation of ULK1, thereby suppressing autophagy initiation. To identify therapeutic targets, we performed a transcriptome-based drug screen using gene signature reversal, which nominated copanlisib, an FDA-approved Pi3k inhibitor, as a promising candidate. Functional validation in human pluripotent stem cell-derived neuromuscular organoids demonstrated that copanlisib reactivates autophagy via restoration of ULK1 activity. Together, our findings uncover a mechanistic link between extracellular matrix dysregulation and impaired autophagy in GNE myopathy and highlight copanlisib as a potential therapeutic strategy.

## Targeting AKT mTORC1 pathway offers hope for GNE myopathy

GNE myopathy is a muscle disorder caused by mutations in the *GNE* gene, leading to muscle weakness. Researchers explored this by using stem cells and mouse cell models to study the disease. They found that a process called autophagy is disrupted in GNE myopathy due to overactive mTORC1 signaling, which normally regulates cell growth and metabolism. The team used a drug screen to find potential treatments and identified copanlisib, an FDA-approved drug, as a promising candidate. Copanlisib was shown to restore autophagy in cell models by inhibiting the PI3K pathway, which is linked to mTORC1 activation. This suggests that targeting this pathway could be a new treatment strategy for GNE myopathy. The study highlights the potential of copanlisib as a therapy and suggests that further research could lead to new treatments for patients with this condition.

This summary was initially drafted using artificial intelligence, then revised and fact-checked by the author.

## Introduction

GNE myopathy (OMIM #605820) is a progressive muscle disorder caused by loss-of-function mutations in the *GNE* gene, which encodes the bifunctional enzyme glucosamine (UDP-*N*-acetyl)-2-epimerase/*N*-acetylmannosamine kinase, essential for sialic acid (SA) biosynthesis^1–3^. Clinically, the disease is characterized by progressive muscle weakness and atrophy, with symptom onset most commonly occurring in early adulthood, typically in the third decade of life and less frequently in childhood or the fifth decade^4^. Although therapeutic trials using SA or its precursors have been conducted, these approaches have shown limited efficacy^5–7^, underscoring the complexity of the underlying pathophysiology. While *GNE* mutations are well established as the genetic cause, how reduced SA biosynthesis translates into progressive myofiber degeneration remains poorly understood.

Due to the scarcity of relevant cellular models, previous studies have relied on GNE-depleted cell lines^8^ or ectopic expression of mutant *GNE* alleles^9^. However, complete *Gne* knockout (KO) in mice is embryonically lethal^10^, and knock-in mouse models recapitulating human mutations have shown only partial phenotypic overlap^11–15^. More recently, patient-derived induced pluripotent stem (iPS) cells have been differentiated into myoblasts exhibiting key pathological features of GNE myopathy, such as hyposialylation and defective autophagy^16^. In particular, isogenic human pluripotent stem (hPS) cell models generated via base editing allow systematic comparison of multiple *GNE* mutations, enabling the identification of mutation-specific phenotypes, transcriptomic signatures and drug responses^17^.

Autophagy plays an essential role in skeletal muscle homeostasis by recycling damaged organelles and proteins, especially under conditions of nutrient scarcity or metabolic stress^18–20^. This process is tightly regulated by nutrient-sensing pathways, including mTORC1 and AMPK, which converge on ULK1, a key autophagy-initiating kinase^21^. AMPK promotes autophagy by phosphorylating ULK1 at Ser317 and Ser777 during energy stress, while mTORC1 inhibits autophagy through phosphorylation of ULK1 at Ser757, disrupting its interaction with AMPK^22^. Dysregulation of autophagy is a known contributor to muscle atrophy and dystrophy. In skeletal muscle, impaired autophagy leads to reduced regenerative capacity due to muscle stem cell depletion^23–25^, a major concern in energy-demanding tissues^26^. Defective autophagy is also implicated in the formation of rimmed vacuoles, a hallmark of GNE myopathy^5,27^.

In this study, we demonstrate that autophagy-related gene signatures are markedly altered in GNE myopathy models derived from both hPS cells^17^ and the C2C12 myoblast cell line. Transcriptome analysis predicted that constitutive mTOR activation underlies the autophagy defect. To identify potential therapeutic candidates, we performed a connectivity map (CMap)-based drug screen and identified copanlisib, a US Food and Drug Administration (FDA)-approved Pi3k inhibitor, as a top candidate with a gene signature inversely correlated to GNE pathology. We validated that copanlisib restores autophagy in both C2C12 cells and hPS cell-derived neuromuscular organoids (NMOs) by reactivating the ULK1 pathway. These results suggest a mechanistic link between mTOR hyperactivation and defective autophagy in GNE myopathy and support copanlisib as a promising therapeutic approach.

## Material and methods

### Cell culture

C2C12 murine myoblasts were cultured in Dulbecco’s modified Eagle medium (DMEM) supplemented with 10% fetal bovine serum (FBS) and 50 μg/ml gentamicin in 5% CO_2_ at 37 °C. Cells were passaged when they reached 60–70% of confluence. They were washed with Dulbecco’s phosphate-buffered saline (DPBS) and detached with 0.25% trypsin–EDTA. Detached cells were rinsed with DMEM/F-12 medium and plated on a tissue culture dish. For glucose starvation, cells were washed twice with DPBS and cultured in DMEM without glucose, while for the general starvation condition, cells were washed twice with DPBS and cultured in Earle’s Balanced Salt Solution (EBSS). Human embryonic stem (hES) cells WA09 (H9; WiCell Research Institute) and the isogenic I329T mutant line were used in this study. The I329T hES cell line was generated from WA09 via base editing, as previously described^17^. hES cells were cultured at 37 °C in mTeSR1 medium (STEMCELL Technologies, #85850) on Corning Matrigel-coated culture dishes (#354277). Cells were dissociated using Versene solution (Thermo Fisher Scientific) upon reaching 70–80% confluency, passaged every 4 days and maintained in mTeSR1 medium, which was replaced daily. For improved cell attachment, 10 μM Y-27632 (Gibco) was added as needed.

### Bulk RNA-seq processing and analysis

Low-quality bases and adapter sequences bases were trimmed using TrimGalore (www.bioinformatics.babraham.ac.uk/). The trimmed reads were aligned to the mouse (GRCm39) or human (GRCh38) reference genome, as appropriate, using STAR (v2.7.3a). Transcript abundance, including expected read counts and transcripts per million, was quantified using RSEM (v1.3.3) with the corresponding gene annotations. Differential expression analysis was performed using the DESeq2 R package (v1.34.0). Raw read counts were normalized using the median of ratios method implemented in DESeq2. Differentially expressed genes (DEGs) were defined as those with an absolute log_2_ fold change (|log_2_FC|) >1 and an adjusted *P* value <0.05. The BAM files and preprocessed data are available available in the Gene Expression Omnibus (https://www.ncbi.nlm.nih.gov/geo/) under accession number GSE278862.

### Differential expression analysis and functional enrichment

To analyze differential gene expression between the control (normal, wild type (WT)) and disease (I329T, *Gne* KO) groups, we used Wald tests and moderated *t*-tests, implemented in the R packages DESeq2 (v3.15) and limma (v3.54), respectively. In addition, for the clinical data, DEGs between normal and affected samples were identified using moderated *t*-tests within the limma package. DEGs were identified using cutoffs of a false discovery rate (FDR)-adjusted *P* value <0.01 and an absolute fold change >2. To investigate the signaling pathways associated with these DEGs, a hypergeometric test was performed using gene sets from MSigDB Hallmark, Reactome, Kyoto Encyclopedia of Genes and Genomes, and Gene Ontology (GO). The resulting FDR-adjusted *P* values were transformed into −log_10_(FDR) values to score the significance of each pathway. In addition, gene set enrichment analysis (GSEA) was conducted to identify pathways significantly overrepresented at the top or bottom of ranked gene lists obtained from the differential expression analysis between different comparison groups (for example, I329T versus normal, *Gne* KO versus WT and PC1 high versus PC1 low). The GSEA was performed using the R package fgsea with the following parameters: minSize = 10, maxSize = 500 and nperm = 100,000. The pathway score was defined as the product of −log_10_(FDR) and the sign of the normalized enrichment score (NES), reflecting both statistical significance and the direction of the enrichment.

### Quantitative GO term analysis and pathway categorization

To quantitatively compare the relative contribution of biological processes highlighted by transcriptomic analyses, GO enrichment results were further analyzed beyond simple pathway counts. GO biological process (BP) terms were first ranked by FDR, and the top 110 enriched GO terms were selected for downstream analysis. To restrict the analysis to biologically meaningful pathways, only GO terms with an absolute NES (|NES|) ≥1 were retained. To account for both enrichment strength and statistical significance while minimizing bias arising from unequal numbers of GO terms per category, we calculated an integrated enrichment score for each biological category. Specifically, for each category, an integrated score was computed as the mean value of |NES| × –log_10_(FDR) across all GO terms assigned to that category. This metric capture both the magnitude and robustness of pathway enrichment without relying on raw GO term counts. GO terms were manually grouped into major biological categories, including cell cycle/metabolism, stress response, protein homeostasis, autophagy and others, based on functional annotations. The resulting category-level scores were used for comparative visualization and interpretation of pathway-level perturbations across GNE-deficient models.

### Integration of existing transcriptome data with produced transcriptome data

To integrate the existing transcriptome data with our newly generated transcriptome data, we performed principal component analysis (PCA). First, we identified 2,758 DEGs (adjusted *P* value <0.05) between normal and I329T. From these DEGs, we selected the top 500 genes with the highest variance across the normal, V727M, R160Q, I588T and I329T samples. Among these, 413 overlapping genes were identified. Using the expression values of these 413 genes, we performed PCA analysis on both the five existing samples (WT, V727M, R160Q, I588T and I329T) and our newly generated C2C12 *Gne* WT and KO samples by using their normalized counts. In addition, we calculated the Pearson correlation of the expression values of the 413 genes to assess the relationship between our generated samples and the existing ones.

### Single-cell data analysis

Using the SEURAT package, we identified markers for each of the two control and the single GNE samples at the late induced myogenic precursor cell (iMPC) stage from PRJNA739168 by applying the FindMarkers function. To assign pathway activity scores for each sample, we performed gene set variation analysis (GSVA) using the R package gsva, with the log_2_FC values of the respective markers as input. To further investigate the signaling pathways associated with these markers, we conducted a hypergeometric test using the MSigDB Wikipathways gene sets. The resulting FDR-adjusted *P* values were transformed into −log_10_(FDR) values to quantify the significance of each pathway.

### Autophagosome/autolysosome imaging

Both WT and *Gne* KO C2C12 myoblasts were transfected with pDEST-CMV mCherry-GFP-LC3B (#123230, addgene) using Lipofectamine 3000 (Thermo Fisher Scientific) according to the manufacturer’s protocol. When the cells reached approximately 70% confluence, 3 µg of plasmid DNA was used for transfection. In the case of glucose deprivation, cells were washed three times with DPBS and changed to DMEM, no glucose (#11966025, Gibco) with 10% FBS and 50 μg/ml gentamicin. TCS SP8 confocal microscope (Leica) was used for imaging samples.

### Identification of potential therapeutics for GNE myopathy using CMap data

We aimed to identify potential drugs that could reverse the effects of GNE myopathy by utilizing CMap data. To achieve this, we used two similarity measures: Pearson correlation and cosine similarity, comparing the logFC values from GNE myopathy models with the drug-induced gene expression signatures found in CMap across various core cell lines. The first dataset comprised level 5 CMap data, which contains drug-induced gene expression signatures for 978 landmark genes across 9 core cell lines: A375, A549, HCC515, HEPG2, MCF7, PC3, VCAP, HT29 and HA1E. To ensure the quality and relevance of the data, we selected only samples with QC_pass = 1 and Pert_time = 24 h, and we limited our analysis to compounds with standardized nomenclature. The second dataset included logFC values for 978 genes derived from four GNE myopathy variants (V727M, R160Q, I588T and I329T), compared with WT controls. Of the 978 genes, 964 were common between the CMap data and the GNE myopathy models, and these overlapping genes were used for the analysis. Drugs with large negative Pearson correlation and cosine similarity values were considered promising candidates for counteracting GNE myopathy. Based on this criterion, we selected the top ten drugs with the most significant negative similarity scores for each cell line. Notably, mTOR and PI3K inhibitors identified among the top ten candidates in MCF7, A549 and PC3 cell lines are summarized in Supplementary Fig. 6e. The full list of these compounds is provided in Supplementary Fig. 6g.

### Generation of NMOs from hPS cells

NMOs were produced from hES cells as previously described^28^. hPS cells were dissociated into single cells using Accutase when cultures reached 70–80% confluency. A total of 1.3–1.5 × 10⁶ cells were counted and seeded onto Matrigel-coated six-well plates. Due to inherent differences in growth rate and clonal heterogeneity, seeding density was optimized for each cell line. On day 0, to mitigate dissociation-induced stress, 10 μM Y-27632 (ROCK inhibitor) was added to the NMO basal medium, which also contained 3 μM CHIR99021 and 40 ng/ml basic fibroblast growth factor. The NMO basal medium consisted of a 1:1 mixture of Advanced DMEM/F12 (supplemented with 1× N2) and Neurobasal medium (supplemented with 1× B27, 2 mM L-glutamine, 75 μg/ml bovine serum albumin fraction V and 0.1 mM 2-mercaptoethanol). On day 1, the medium was refreshed with the same formulation excluding Y-27632, and from day 1 to day 3, media were replaced daily. Neuromesodermal progenitors were generated using this two-dimensional induction protocol. On day 3, neuromesodermal progenitors were dissociated into single cells using Accutase and seeded at 8,000 cells per well into ultralow-attachment 96-well plates. Cell numbers were quantified using a Countess III automated cell counter. Aggregation was initiated by centrifuging the plates at 350*g* for 2 min. For organoid formation (day 0 of three-dimensional culture), each well received 100 μl of NMO basal medium supplemented with 50 μM Y-27632, 10 ng/ml basic fibroblast growth factor, 2 ng/ml insulin-like growth factor 1 (IGF1) and 2 ng/ml hepatocyte growth factor (HGF). On day 2, 50 μl of medium was removed and replaced with 100 μl of fresh NMO basal medium containing 2 ng/ml IGF1 and 2 ng/ml HGF. On day 4, medium was completely replaced with NMO basal medium without additional growth factors and was subsequently refreshed every two days. On day 10, organoids were transferred to 60-mm ultralow-attachment dishes containing 5 ml of NMO basal medium. On day 30, they were further transferred to 100-mm Petri dishes containing 12 ml of medium. Medium was changed every other day. During transfers, pipette tips were trimmed with autoclaved scissors to enable gentle handling of organoids. Starting on day 10, organoids were maintained on an orbital shaker at 75 rpm to promote maturation.

### Immunocytochemistry of organoids

Organoids were collected at designated time points (day 25 and day 50 of differentiation), washed once with DPBS and fixed in 4% paraformaldehyde at 4 °C for 1–2 h, depending on the culture duration. Following fixation, samples were washed three times with DPBS and incubated in 30% sucrose at 4 °C overnight until organoids fully sank, indicating complete infiltration. Organoids were embedded in Tissue-Tek OCT compound using embedding molds. For cryopreservation, isopentane (2-methylbutane) was partially solidified by placing a metal container in a Styrofoam box filled with liquid nitrogen. Once the isopentane turned opaque, embedding molds were rapidly immersed and frozen. Frozen blocks were stored at –80 °C until sectioning. Cryosections were cut at 10 μm thickness using a Leica cryostat set to –19 °C and mounted onto glass slides. Before staining, slides were brought to room temperature and washed with 0.3% Triton X-100 in DPBS to remove residual OCT compound. Sections were then blocked for 1 h at room temperature using either 5% normal goat serum or 4% bovine serum albumin in DPBS. Primary antibodies were diluted in the same blocking buffer and applied to sections for overnight incubation at 4 °C. Antibodies included anti-LC3B (1:100; Cell Signaling Technology, #2775S), SNA lectin (1:400; Vector Laboratories, FL-1301), anti-MYOD (1:400; Cell Signaling Technology, #13812S), anti-Fast myosin heavy chain (MyHC; 1:200; Sigma-Aldrich, M1570) and anti-β-tubulin III/Tuj1 (1:500; Biozol, GTX129913-25). The following day, slides were washed three times with DPBS and incubated with fluorophore-conjugated secondary antibodies for 1–1.5 h at room temperature. After three additional washes with DPBS, nuclei were counterstained with 4′,6-diamidino-2-phenylindole (DAPI; 1 μg/ml) for 10 min and washed once more. Finally, sections were mounted using ProLong Gold Antifade Reagent.

### Statistical analysis

The graphical and quantitative data were presented as mean ± standard deviation. Statistical significance among the three groups and between groups was determined using one-way or two-way analysis of variance (ANOVA) following Tukey’s post-hoc test and Student’s *t-*test, respectively. Statistical analysis was performed with GraphPad Prism 8 software (https://www.graphpad.com/scientific-software/prism/). Significance was assumed for *P* < 0.05 (*), *P* < 0.01 (**), *P* < 0.001 (***) and *P* < 0.0001 (****).

## Results

### Transcription analysis of isogenic GNE myopathy models derived from hPSCs

**Fig. 1:**
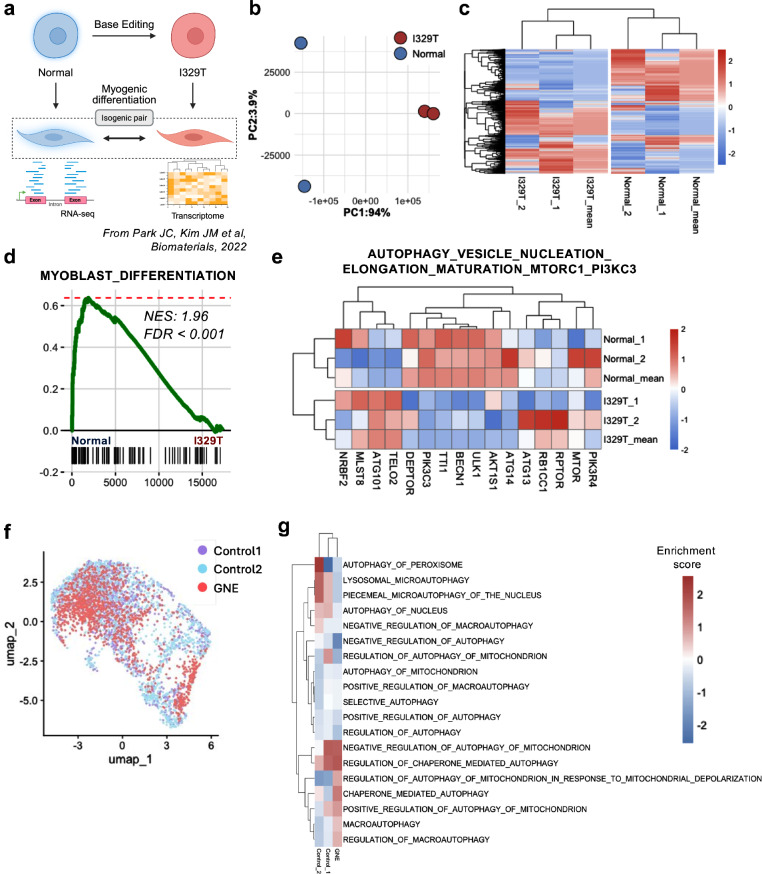
Transcriptional analysis from isogenic GNE myopathy models derived from hPS cells. **a**, Diagram of normal hPS cells, an isogenic *GNE* I329T hPSC line (I329T mutation introduced by ABE), myogenic differentiation into paired GNE myoblasts (normal and I329T) and subsequent bulk RNA-seq and transcriptome analysis. **b**, PCA of normal (blue) and I329T (red) myoblasts. **c**, Hierarchical clustering heatmap of gene expression profiles in normal and I329T cells. **d**, GSEA plot displaying the enrichment for the gene set ‘MYOBLAST_DIFFERENTIATION’ in gene rank based on differential expression between the I329T versus normal. **e**, Heatmap of gene expression patterns for the ‘AUTOPHAGY VESICLE NUCLEATION ELONGATION MATURATION MTORC1 Pi3kC3’ gene set in normal and I329T cells. **f**, Uniform Manifold Approximation and Projection (UMAP) of single-cell data from control 1, control 2 and GNE myopathy patient iPS cells differentiated into myoblasts. **g**, Heatmap of GSVA enrichment scores for autophagy-related gene set in myoblasts differentiated from control 1, control 2 and GNE myopathy patient samples.

Previously, we reported that myoblasts derived from hPS cells harboring the *GNE* I329T mutation—located in the epimerase domain and frequently observed in Asian populations^29^—exhibited pronounced pathogenic phenotypes compared with their isogenic WT counterparts (hereafter referred to as normal)^17^ (Fig. 1a). Two transcriptomic datasets of *GNE*-mutant hPSC-derived myoblasts, particularly those harboring the I329T mutation (hereafter referred to as I329T), are currently available: (1) hES cell-derived myoblasts (normal versus I329T) generated in our prior study^17^, and (2) GNE patient iPS cell-derived myoblasts (control 1 and control 2 versus GNE) from a previous study (PRJNA739168)^16^ (Supplementary Fig. 1a).

PCA (Fig. 1b) and heatmap visualization (Fig. 1c) of gene expression profiles clearly distinguished I329T cells from normal cells, underscoring their transcriptional divergence. Differential expression analysis further revealed downregulation of genes involved in myogenic differentiation (Fig. 1d) and muscle development (Supplementary Fig. 1b) in I329T cells, consistent with findings from patient-derived iPS cell models^16^.

Given the established link between defective autophagy and GNE myopathy^30^, as reported in patient tissues^31^ and in vitro models^32,33^, we examined gene signatures derived from DEGs between normal and I329T myoblasts. GO BP analysis revealed strong enrichment of autophagy-related gene signatures (Supplementary Fig. 1c), which were visualized as relative expression changes (Fig. 1e and Supplementary Fig. 1d,e).

Notably, a separate myoblast model derived from GNE patient iPS cells (that is, control versus GNE)^16^ (Fig. 1f), similarly showed disruption of autophagy gene signatures (Fig. 1g), reinforcing the notion that autophagy impairment is a key pathogenic feature of GNE myopathy.

### Establishment of Gne-KO myoblasts

**Fig. 2:**
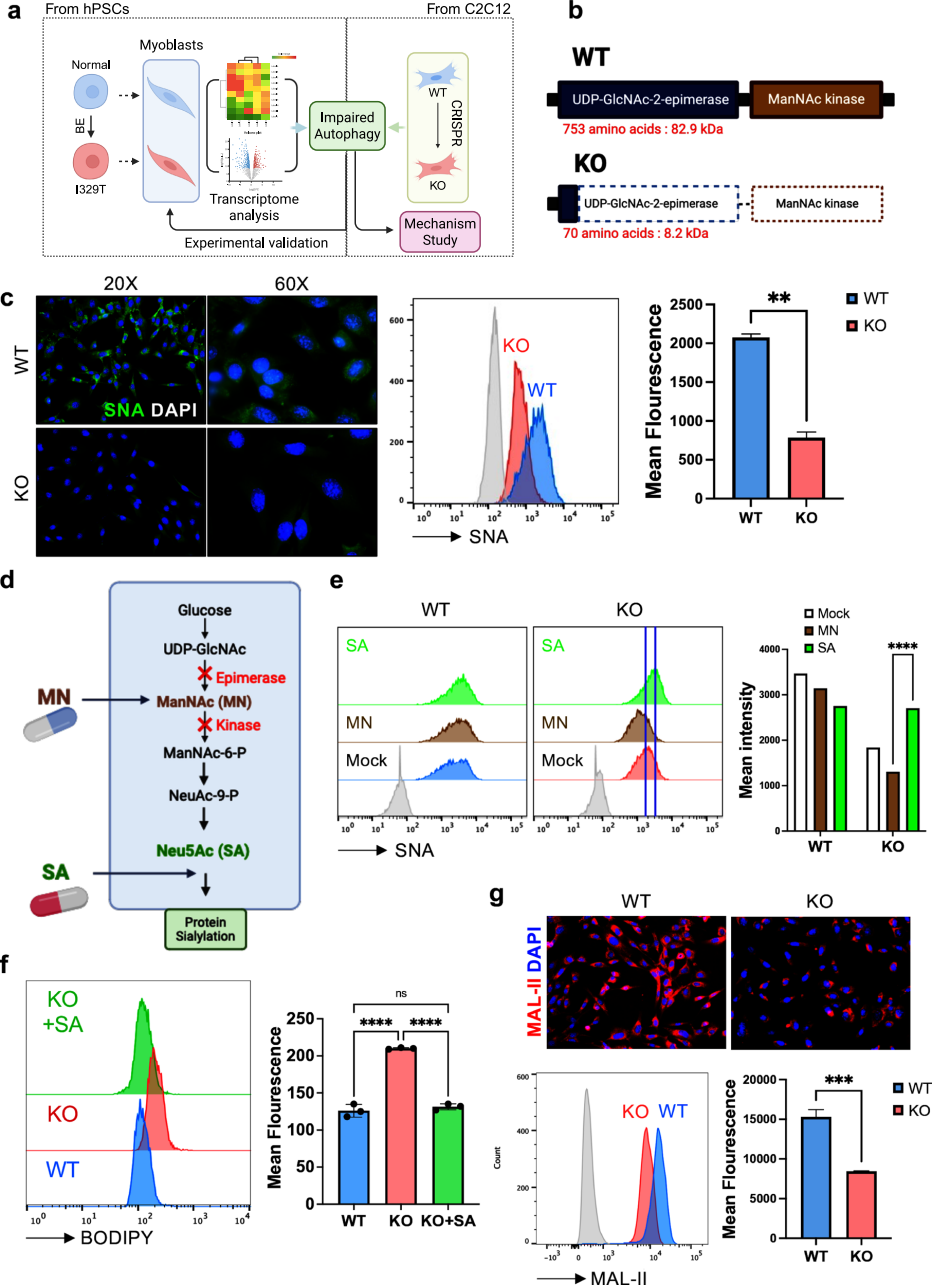
Establishment of *Gne*-KO myoblasts. **a**, Schematic overview of experimental strategies using isogenic pairs of hPS cells (normal and I329T) and C2C12 cells (WT and KO). **b**, Graphical description showing the enzyme domain of both WT and KO cells. Dashed boxes indicate the deleted regions in KO cells. **c**, Immunofluorescence images (left) of WT and KO cells stained with SNA (green) and DAPI (blue). Histogram (middle) and bar graph (right) of mean fluorescence intensity of SNA in WT and KO cells by flow cytometry. The gray peak represents the unstained control. Statistical analysis was performed using unpaired *t*-test (*P* = 0.0022). **d**, Graphical description of the experimental overview for the treatment with *N*-acetylmannosamine (MN) and SA. **e**, Histogram (left) and bar graph (right) of mean fluorescence intensity of SNA in WT and KO cells after treatment with MN and SA. The gray peak represents the unstained control. Statistical analysis was performed using two-way ANOVA (*P* < 0.0001). **f**, Histogram (left) and bar graph (right) of mean fluorescence intensity of BODIPY in WT, KO and KO after treatment with SA (1 mM, 5 days). Statistical analysis was performed using one-way ANOVA (*P* < 0.0001). **g**, Immunofluorescence images (top) of WT and KO cells stained with MAL-II (red) and DAPI. Histogram and bar graph (bottom) of mean fluorescence intensity of MAL-II in WT and KO cells by flow cytometry. The gray peak represents the unstained control. Statistical analysis was performed using unpaired *t*-test (*P* = 0.0002).

To complement transcriptome-based predictions (Fig. 1) and elucidate the mechanism underlying defective autophagy, we sought an alternative to the labor-intensive differentiation of GNE myoblasts from hPS cells. A stable and reproducible cell model that recapitulates key pathogenic features—namely hyposialylation and impaired autophagy—would provide a robust platform for biochemical validation and mechanistic studies (Fig. 2a). To this end, we used the mouse C2C12 myoblast cell line (WT) to generate an isogenic *Gne*-KO model, hereafter referred to as WT and KO to clearly distinguish this system from the human hPS cell-derived models used elsewhere in the study. Using CRISPR–Cas9-mediated genome editing, an indel mutation was introduced into exon 2, resulting in a premature stop codon (Supplementary Fig. 2a,b). Thus, KO cells express a truncated Gne protein lacking both the epimerase and kinase domains compared with WT (Fig. 2b).

Loss of Gne function in KO cells was confirmed by pronounced hyposialylation, visualized via Sambucus nigra agglutinin (SNA) lectin staining, which specifically detects terminal α (2,6)-linked SA residues (Fig. 2c). As reduced sialylation is closely associated with disease severity in GNE myopathy^17^, possibly through induction of oxidative stress^34^, supplementation with *N*-acetylmannosamine (ManNAc), SA, or their metabolic intermediates has been explored as a therapeutic strategy^35^. However, clinical outcomes have been inconsistent^6,36,37^.

ManNAc supplementation selectively rescues protein sialylation in epimerase-domain mutants, while SA supplementation exerted broader effects (Fig. 2d). In our KO cells, which lack both epimerase and kinase activity, SA restored sialylation, but ManNAc—requiring functional ManNAc kinase—did not (Fig. 2e). Supporting previous reports of elevated glycosphingolipid levels in *GNE* knock-in mice and blood plasma from patients with GNE myopathy^38^, 4,4-difluoro-4-bora-3a,4a-diaza-s-indacene (BODIPY) staining, which reflects lipid accumulation in sphingolipid- and glycosphingolipid-enriched compartments^39^, was markedly increased in KO cells and reduced upon SA treatment (Fig. 2f). Hyposialylation in KO cells was further validated using *Maackia amurensis* lectin II (MAL II), which preferentially binds α(2,3)-linked SAs (Fig. 2g).

### Defective autophagy in *Gne*-KO cells upon nutrient deficiency

**Fig. 3:**
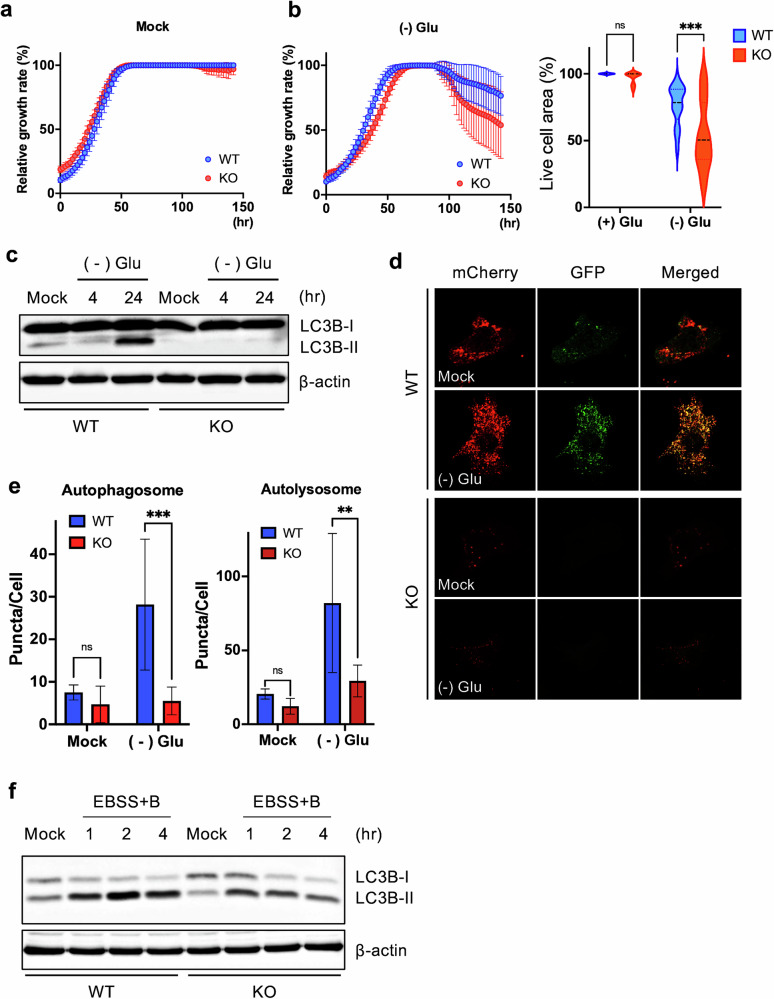
Defective autophagy in *Gne*-KO cells upon nutrient deficiency. **a**, Graph showing live cell area of WT and KO cells measured every 2 h over 142 h under mock ((+) Glu) condition. **b**, Graph (left) showing live cell area measured every 2 h over 142 h under glucose deprivation ((−) Glu) condition and violin plot (right) showing live cell area at 142 h. Statistical analysis was performed using two-way ANOVA (*P* = 0.0008). **c**, Immunoblot analysis of WT and KO cells showing LC3(I/II) levels under mock conditions and after 4 and 24 h of glucose starvation. **d**, Immunofluorescence images of WT and KO cells transiently expressing mCherry-GFP-LC3 under mock conditions and 24 h glucose starvation conditions. **e**, Bar graphs showing the number of puncta per cell for both autophagosomes and autolysosomes. Puncta expressing both GFP and mCherry, appearing yellow, were counted as autophagosomes, while puncta expressing only mCherry were counted as autolysosomes. Statistical analysis was performed using two-way ANOVA (*P* = 0.0001, 0.0013). **f**, Immunoblot analysis of WT and KO cells showing LC3(I/II) levels under mock conditions and after 1, 2 and 4 h of EBSS starvation with Bafilomycin A1.

Despite the clear evidence of hyposialylation in *Gne*-KO cells (Fig. 2), overt phenotypic changes indicative of myopathy were less apparent under standard culture conditions, as reflected by the comparable proliferation rates of WT and KO cells (Fig. 3a). However, under glucose deprivation—a key metabolic stress condition highly relevant to skeletal muscle physiology, including energy metabolism^40^ and autophagy regulation^41^—KO cells exhibited significantly increased susceptibility to cell death (Fig. 3b). Given the high metabolic demands of muscle tissue and its reliance on a constant energy supply, basal autophagy plays a crucial role in maintaining energy homeostasis. We therefore hypothesized that the autophagy impairment predicted by transcriptome analysis (Fig. 1) could underlie the heightened vulnerability of KO cells during glucose starvation, as observed in Fig. 3b.

Indeed, defective autophagy under glucose-starved conditions [(−) Glu] was confirmed by a marked reduction in LC3-phosphatidylethanolamine conjugate (LC3-II) levels in KO cells, indicating impaired autophagosome formation (Fig. 3c). This defect was further validated using an mCherry-GFP-LC3 reporter construct, which distinguishes autophagosomes (yellow puncta: GFP^+^ mCherry^+^) from autolysosomes (red puncta: GFP^–^ mCherry^+^)^42^ (Fig. 3d). Under glucose withdrawal, WT cells displayed a robust increase in both autophagosomes and autolysosomes, whereas KO cells showed a significant reduction in both compartments (Fig. 3e).

To further assess autophagic flux, we induced autophagy using EBSS, which lacks amino acids and serum, in the presence of bafilomycin A1, a lysosomal inhibitor that blocks autophagosome–lysosome fusion^43^. Under these conditions, LC3B-II accumulation was substantially reduced in KO cells compared with WT cells (Fig. 3f), consistent with results from the mCherry-GFP-LC3 reporter assay (Supplementary Fig. 3). Collectively, these findings demonstrate that autophagosome formation and autophagic flux are disrupted in KO cells under nutrient-deprived conditions. Notably, autophagy dysfunction has been proposed as a pathogenic mechanism contributing to rimmed vacuole formation in GNE mouse models^15^, and similar defects have been independently reported in various cell-based models^16,32,33^. These findings support the *Gne*-KO cell line as a reliable and practical platform for investigating the mechanistic basis of autophagy impairment in GNE myopathy, offering a scalable alternative to the labor-intensive generation of myoblasts from GNE hPS cells.

### Upregulated Pi3k/AKT/mTOR signaling in *Gne*-KO cells

**Fig. 4:**
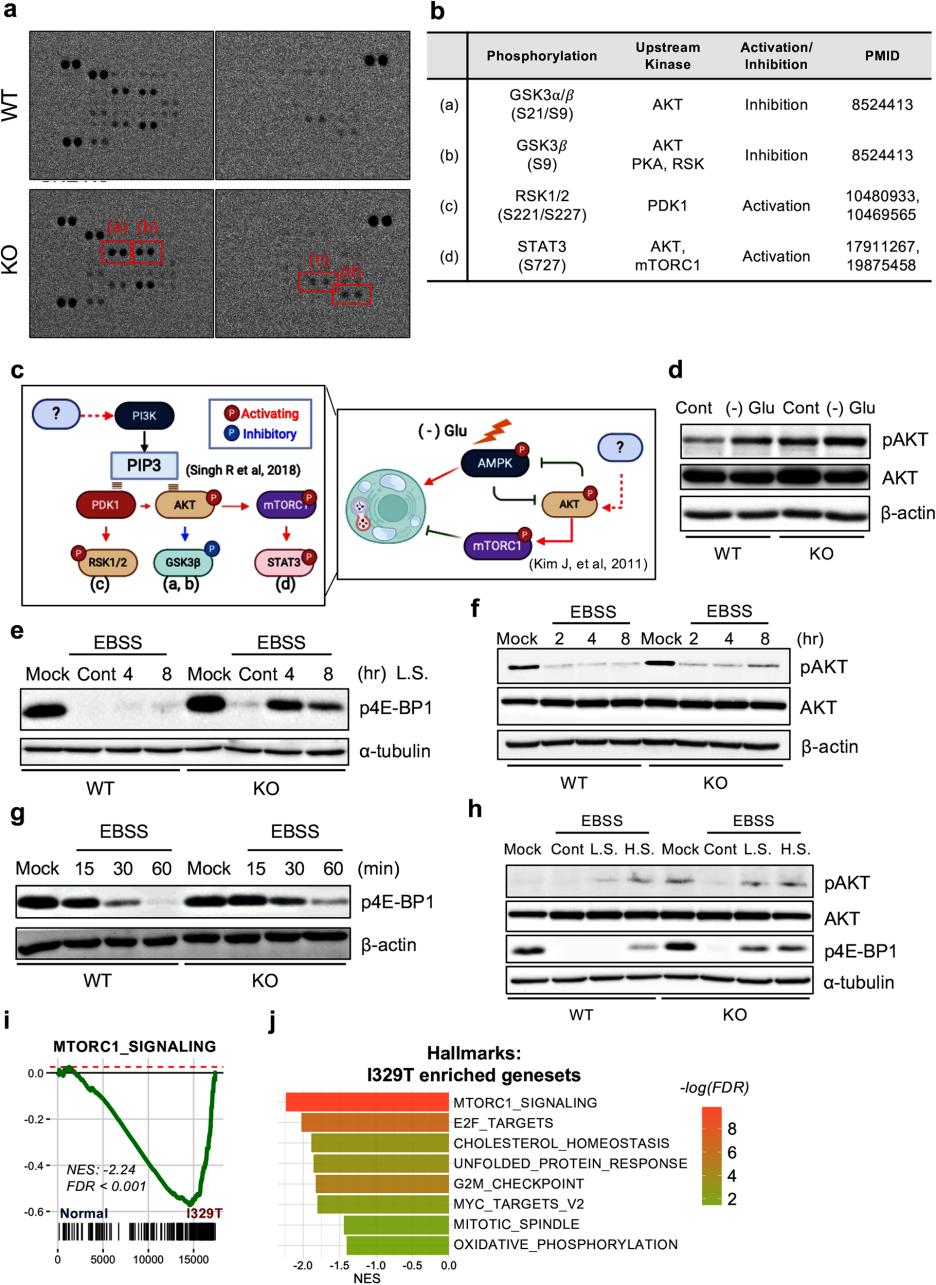
Upregulated Pi3k/AKT/mTOR signaling in *Gne*-KO cells. **a**, Representative image of immunoassay-based phospho-kinase proteome profiler in both WT and KO cells, highlighting (a) GSK3α/*β* (S21/S9); (b) GSK3*β* (S9); (c) Rsk1/2 (S221/S227); and (d) Stat3 (S727) with red boxes. **b**, Table showing the roles of phosphorylation and their corresponding upstream kinases. **c**, Graphical description of the hypothesis regarding the signaling pathway, showing protein phosphorylation and interactions between upstream proteins. **d**, Immunoblot analysis of WT and KO cells showing total AKT and AKT phosphorylation at the serine 473 residue (S473) under mock conditions and 24 h glucose starvation conditions. **e**, Immunoblot analysis of WT and KO cells showing 4E-BP1 phosphorylation at the serine 65 residue (S65) under mock conditions and after 0, 4 and 8 h of EBSS treatment. Mock: DMEM with 10% FBS; Cont.: EBSS without FBS; L.S.: EBSS with 1% FBS. **f**, Immunoblot analysis of WT and KO cells showing total AKT and AKT(S473) phosphorylation under mock conditions and after 0, 2, 4 and 8 h of EBSS treatment. **g**, Immunoblot analysis of WT and KO cells showing 4E-BP1(S65) phosphorylation under mock conditions and after 0, 15, 30 and 60 min of EBSS treatment. **h**, Immunoblot analysis of WT and KO cells showing total AKT and AKT (S473) and 4E-BP1 (S65) phosphorylation under mock conditions and after 4 h of EBSS treatment under low- and high-serum conditions. L.S.: 1% FBS; H.S.: 10% FBS. **i**, GSEA plots displaying the enrichment for the gene set ‘MTORC1_SIGNALING’ in gene rank based on differential expression between the I329T versus normal. **j**, Bar graph illustrating the enrichment score for MSigDB hallmark gene sets in DEGs of I329T cells compared with normal cells, quantified through GSEA.

To investigate upstream mechanisms contributing to impaired autophagosome formation in the *Gne*-KO cells, we performed a phospho-kinase proteome profiling assay. Among the 44 phosphoproteins assessed, we observed increased phosphorylation of GSK3α/β (Ser9 and Ser9/21), RSK1/2 (Ser221/227) and STAT3 (Ser727) in KO cells (Fig. 4a). The functions of these phosphorylation events and their upstream kinases are summarized in Fig. 4b. Notably, increased inhibitory phosphorylation of GSK3β (Ser9)—a well-established downstream event of AKT activation—was observed in KO cells (Fig. 4a, (a) and (b)). This finding was further supported by reduced phosphorylation of CREB (Ser129), a direct downstream target of active GSK3β (Supplementary Fig. 4a). Together, these data indicate aberrant activation of the PI3K–PDK1–AKT signaling axis in KO cells, as summarized schematically in Fig. 4c.

Notably, increase of active phosphorylation of AKT has also been reported in human *GNE*-knockdown cells^8^, further supporting these signaling alterations in the KO model. Given that AMPK and AKT–mTORC1 have opposing roles in autophagy regulation^22^, the defective autophagy observed under glucose or nutrient deprivation in KO cells (Fig. 3) may result from reduced AMPK activation and/or enhanced AKT signaling. Consistently, phosphorylated AKT remained elevated in KO cells even during glucose starvation, with comparable total AKT levels (Fig. 4d). To evaluate mTORC1 activity, we examined phosphorylation of 4E-BP1, a direct downstream target of mTORC1. While phospho–4E-BP1 levels were similar between WT and KO cells under 10% serum, KO cells maintained elevated phosphorylation even under low serum (1%) conditions (Fig. 4e). Normally, nutrient withdrawal (for example, EBSS treatment) suppresses AKT–mTORC1 signaling and activates AMPK. However, in KO cells, EBSS-induced reductions in phospho-AKT (Fig. 4f) and phospho–4E-BP1 (Fig. 4g) were markedly blunted. Moreover, AKT and mTORC1 signaling persisted even in EBSS with low serum (Fig. 4h), suggesting that KO cells are predisposed to hyperactivation of this pathway.

To determine whether these findings reflect transcriptional alterations seen in human GNE myopathy, we revisited gene expression data from normal versus I329T, hES cell-derived myoblasts. GSEA confirmed upregulation of mTORC1 signaling (Fig. 4i,j). In parallel, single-cell RNA sequencing (RNA-seq) analysis of myogenic progenitors from two control lines (control 1 and control 2) and one GNE patient iPS cell line (GNE) revealed distinct clustering: clusters 1, 5 and 7 were enriched in control cells, while clusters 0 and 8 were predominant in GNE samples (Supplementary Fig. 4b,c). Notably, cluster 8 showed enrichment for focal adhesion and PI3K–AKT–mTOR signaling pathways (Supplementary Fig. 4d), whereas control-enriched clusters were associated with muscle contraction pathways (Supplementary Fig. 4e). Together, these data demonstrate that aberrant PI3K–AKT–mTORC1 activation observed in the C2C12-KO model (Fig. 4) closely parallels transcriptomic features of human GNE myopathy across both hES cell-derived (normal versus I329T) and hiPS cell-derived (control versus GNE) myoblast models, supporting its utility as a mechanistic system.

### High focal adhesion by ECM production for AKT activation in *Gne*-KO cells

**Fig. 5:**
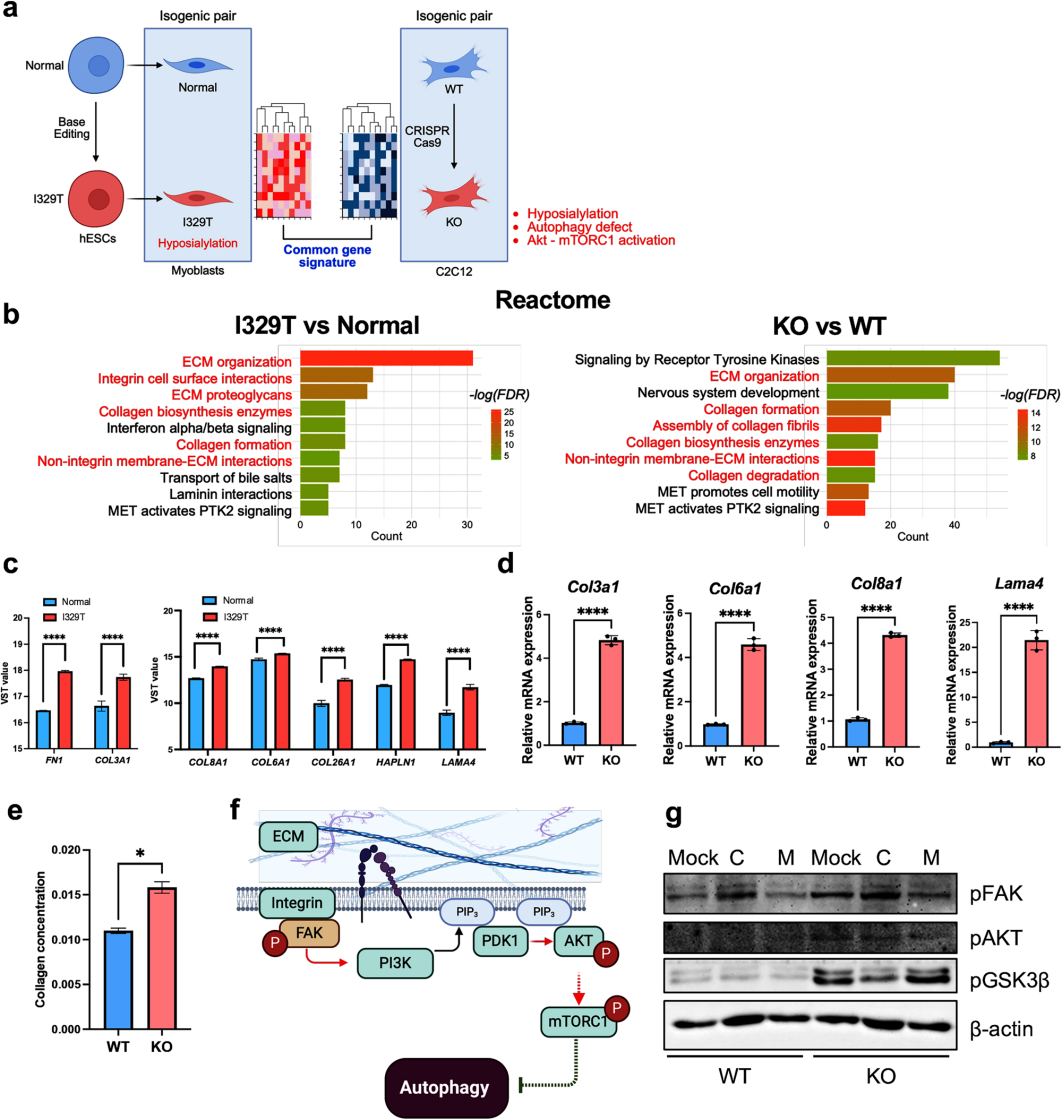
High focal adhesion by ECM production for AKT activation in *Gne*-KO cells. **a**, Graphical description comparing the common gene signatures of two isogenic pairs generated from hES cells (H9) and C2C12 myoblasts. **b**, Enriched MSigDB Reactome gene sets for upregulated genes in I329T cells compared with normal cells (left) and KO cells compared with WT cells (right). **c**, Variance-stabilized (VST) RNA-seq expression values of multiple ECM protein-encoding genes from normal and I329T myoblasts. **d**, Real-time polymerase chain reaction (PCR) data of multiple ECM protein-encoding genes from WT and KO cells. Statistical analysis was performed using unpaired *t*-test (*P* < 0.0001). **e**, Concentration of soluble collagen secreted by WT and KO cells after 3 days of seeding on a 100-mm dish. Statistical analysis was performed using unpaired *t*-test (*P* = 0.0104). **f**, Graphical description of ECM-mediated signal transduction on the Pi3k–AKT–mTOR axis and autophagy. **g**, Immunoblot analysis of WT and KO cells showing phosphorylation of pFAK (Y576/577), pAKT (S473) and pGSK3β (S9) on a normal culture dish (Mock), collagen-coated dish (C) and Matrigel-coated dish (M). hESCs, hES cells.

We identified defective autophagy in *Gne*-KO cells (Fig. 3), consistent with predictions from transcriptome analyses of two independent hPSC-based disease models (Figs. 1 and 3). This defect was accompanied by pronounced hyposialylation (Fig. 2) and aberrant activation of the AKT–mTORC1 signaling axis, which persisted even under serum-deprived conditions (Fig. 4). To investigate the potential drivers of this atypical AKT activation, we analyzed transcriptome datasets from both isogenic hES cell-derived myoblasts (normal and I329T) and C2C12 cells (WT and KO) (Fig. 5a). PCA revealed two distinct clusters based on the presence or absence of pathogenic phenotypes (Supplementary Fig. 5a). KO cells clustered closely with pathogenic hES cell-derived mutants (R160Q, I588T and I329T), while WT cells clustered with the normal and V727M groups—the latter known to retain near-normal SA synthesis^17^ (Supplementary Fig. 5b).

Given that the I329T variant exhibits the most severe pathogenicity^17^, we compared DEGs between normal and I329T myoblasts with DEGs between KO and WT cells. Notably, both datasets showed consistent enrichment of gene signatures related to collagen production, integrin signaling, and the extracellular matrix (ECM), as revealed by Reactome pathway analysis (Fig. 5b), as well as GO terms for molecular function (Supplementary Fig. 5c), BP (Supplementary Fig. 5d) and cellular component (Supplementary Fig. 5e). Specifically, several ECM-related genes, including *FN1*, *COL3A1*, *COL8A1*, *COL6A1*, *COL26A1*, *HAPLN1* and *LAMA4*, were significantly upregulated in I329T mutant myoblasts (Fig. 5c), and corresponding genes such as *Col3a1*, *Col6a1*, *Col8a1* and *Lama4* were likewise elevated in KO cells (Fig. 5d). In support of these transcriptomic findings, KO cells secreted significantly more soluble collagen than WT cells (Fig. 5e). These observations are in line with prior studies reporting ECM remodeling and upregulation of adhesion-related genes in GNE mouse models^13^ and GNE patient muscle tissues^44^. Given that elevated ECM components such as collagen not only contribute to structural remodeling but also activate intracellular signaling cascades^45^, we hypothesized that the high collagen production in KO cells triggers integrin-mediated activation of focal adhesion kinase (FAK), which in turn drives AKT–mTORC1 signaling and disrupts autophagy initiation (Fig. 5f).

Supporting this hypothesis, KO cells exhibited high basal levels of phosphorylated FAK (pFAK), a marker of focal adhesion signaling typically induced by collagen but not by Matrigel. This FAK activation was accompanied by increased phosphorylation of downstream targets AKT and GSK3β (Fig. 5g). Together, these results suggest that excessive ECM production in KO cells promotes activation of the FAK–AKT–mTORC1 axis, which in turn contribute to autophagy suppression.

### High inhibitory phosphorylation of ULK1 in *Gne*-KO cells

**Fig. 6:**
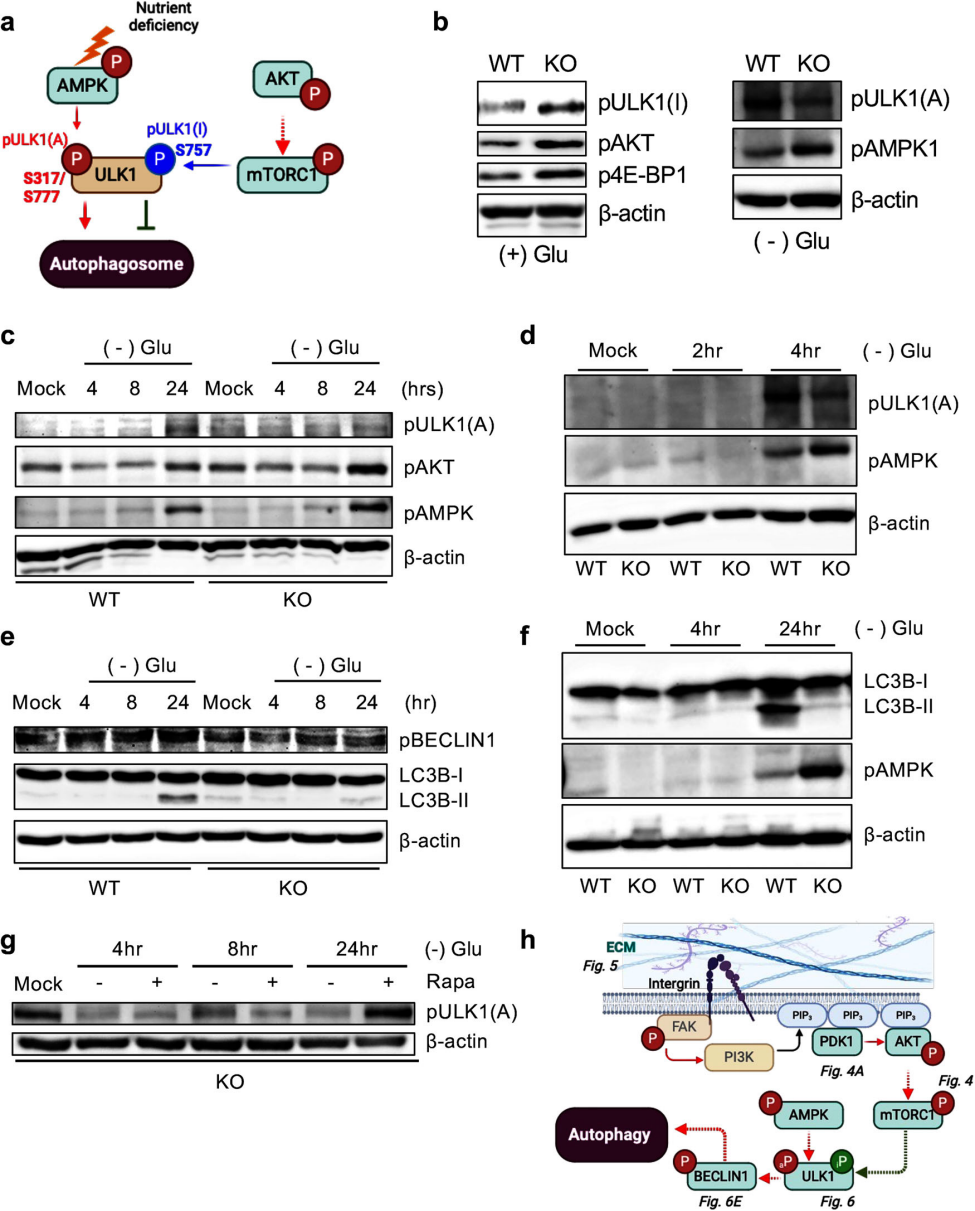
High inhibitory phosphorylation of ULK1 in *Gne*-KO cells. **a**, Graphical description of the AMPK and AKT signaling axis affecting ULK1 complex phosphorylation. S317/S777: activating phosphorylation; S757: inhibitory phosphorylation. **b**, Immunoblot analysis of WT and KO cells showing ULK1(S317: activating), AKT(S473), 4E-BP1(S65) phosphorylation under (+) Glu conditions and ULK1(S757: inhibitory), AMPK(T172) phosphorylation under (−) Glu conditions. **c**, Immunoblot analysis of WT and KO cells showing phosphorylation of ULK1(S317: activating), AKT(S473), and AMPK(T172) under 0 (mock), 4, 8 and 24 h of glucose starvation. **d**, Immunoblot analysis of WT and KO cells showing phosphorylation of ULK1(S317: activating), AMPK(T172) under 0 (mock), 2 and 4 h of glucose starvation. **e**, Immunoblot analysis of WT and KO cells showing phosphorylation of BECLIN1(S15), LC3B(I/II) under 0 (mock), 4, 8 and 24 h of glucose starvation. **f**, Immunoblot analysis of WT and KO cells showing phosphorylation of AMPK(T172), LC3B(I/II) under 0 (mock), 4 and 24 h of glucose starvation. **g**, Immunoblot analysis of KO cells showing phosphorylation of ULK1(S317: activating under 0 (mock), 4, 8 and 24 h of glucose starvation, with or without 100 nM rapamycin. **h**, Graphical description of the signaling axis regulating autophagy initiation by ECM accumulation.

It is well established that UNC51-like kinase 1 (ULK1), a key mammalian homolog of *Saccharomyces* *cerevisiae* Atg1, is tightly regulated by nutrient- and energy-sensing kinase pathways^46^. ULK1 activity is directly modulated by phosphorylation: AMPK, activated under energy stress, promotes autophagy by phosphorylating ULK1 at Ser317 and Ser777 (active phosphorylation: pULK1(A)), whereas mTORC1, activated by growth factors or amino acids, inhibits autophagy by phosphorylating ULK1 at Ser757 (inhibitory phosphorylation: pULK1(I)), thereby disrupting its interaction with AMPK^22^ (Fig. 6a). As depicted in Fig. 6a, the relative balance between these activating and inhibitory phosphorylation events of ULK1 serves as a functional readout of autophagy initiation.

Under standard glucose conditions ((+) Glu), KO cells exhibited elevated basal levels of pULK1(I), along with increased phosphorylation of AKT and 4E-BP1, indicating heightened AKT–mTORC1 signaling. By contrast, during glucose deprivation ((–) Glu), KO cells failed to upregulate pULK1(A) despite robust AMPK activation (Fig. 6b). This failure is probably due to sustained AKT activity, which maintains mTORC1-mediated inhibition of ULK1 and prevents its activation by AMPK^22^. Notably, AKT phosphorylation remained elevated in KO cells as early as 4 h into glucose starvation (Fig. 6c,d), indicating that nutrient-responsive suppression of AKT–mTORC1 signaling is impaired. Consequently, phosphorylation of BECLIN1 at Ser14 (pBECLIN1)—a known ULK1 target associated with autophagy induction^47^—and formation of LC3B-II, a canonical marker of autophagosome formation, were both markedly blunted in KO cells (Fig. 6e), despite evident AMPK activation in KO after glucose deprivation (Fig. 6f). Pharmacological inhibition of mTORC1 using rapamycin restored ULK1 activation under glucose-starved conditions in KO cells, as indicated by increased pULK1(A) level, supporting a role for mTORC1 in autophagy suppression in this context (Fig. 6g). In parallel, AKT phosphorylation (Fig. 4) and ECM–integrin–FAK signaling (Fig. 5), were sustained in KO cells. Together, these data demonstrate that aberrant activation of AKT and ECM–FAK signaling is associated with persistent mTORC1 activity and impaired ULK1-dependent autophagy initiation in *GNE*-deficient cells (Fig. 6h).

### Copanlisib, an FDA-approved drug, for autophagy recovery in GNE myopathy NMOs

To explore potential therapeutic strategies, we first assessed whether restoration of sialylation by SA supplementation—shown to rescue hyposialylation in KO cells (Fig. 2e)—could also correct the autophagy defect. SA treatment resulted in a modest increase in autophagy-related markers in KO cells under standard culture conditions (Supplementary Fig. 6a). However, SA supplementation failed to restore autophagic flux under glucose deprivation, a condition that robustly reveals autophagy impairment in KO cells (Supplementary Fig. 6b). These results indicate that correction of hyposialylation alone is insufficient to rescue defective autophagy, consistent with the limited functional benefits reported for SA replacement therapies in GNE myopathy^6,36,37^.

**Fig. 7:**
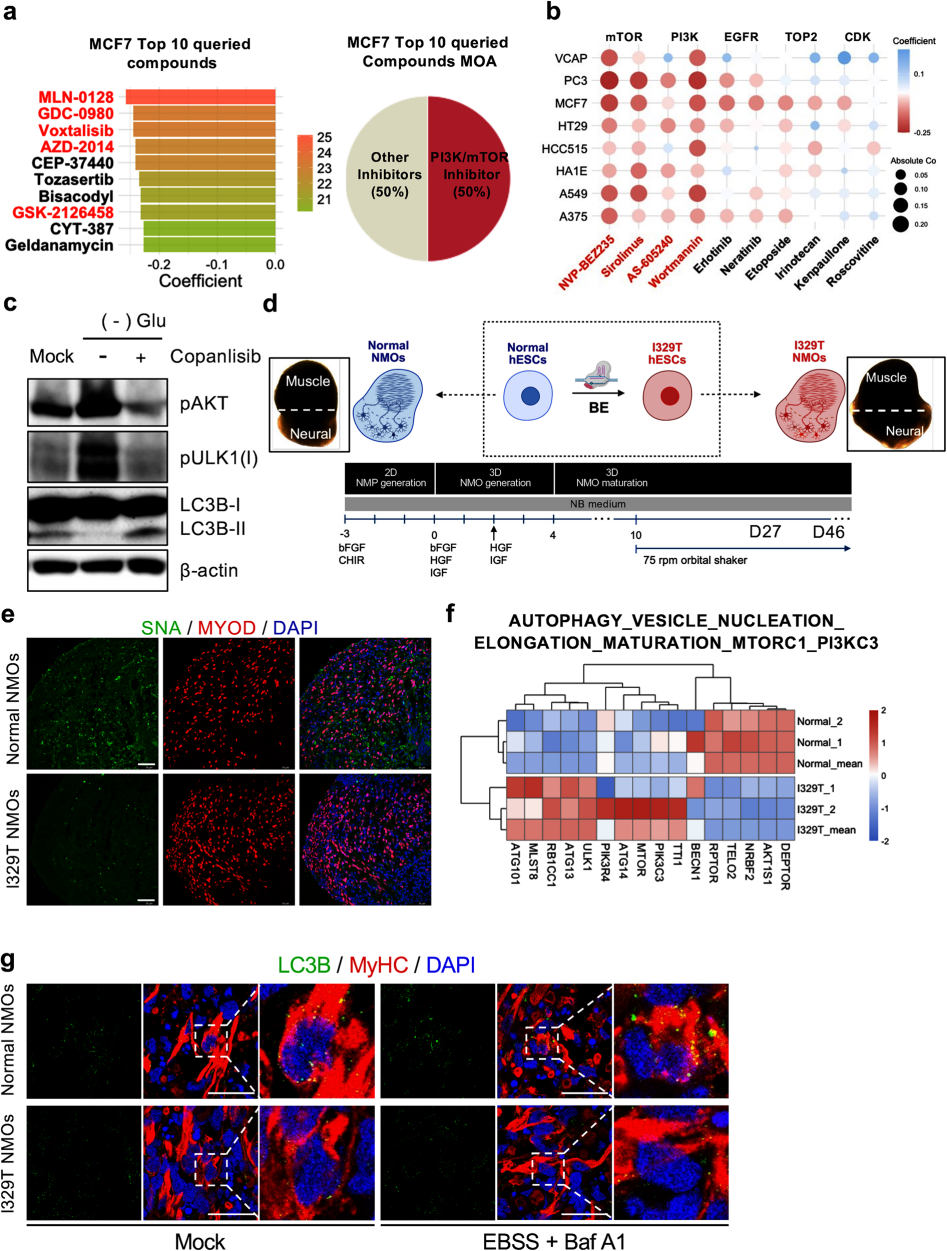

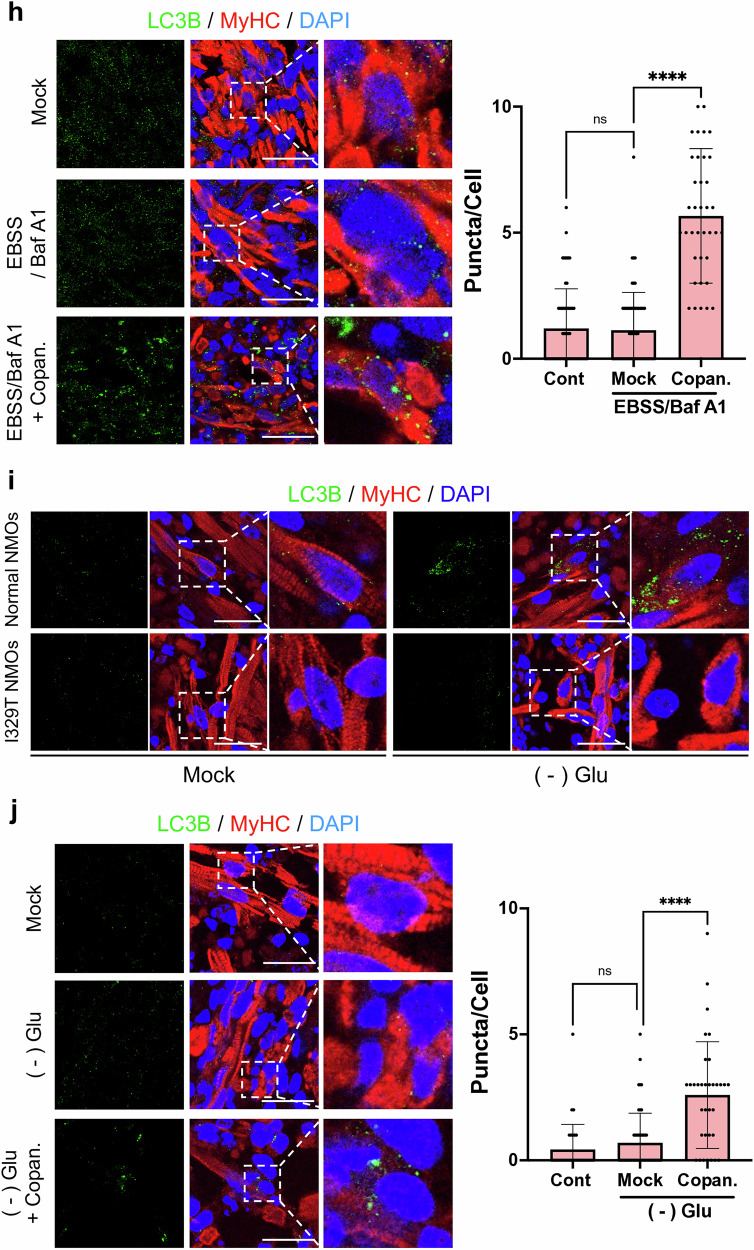
Copanlisib, an FDA-approved drug, for autophagy recovery in GNE myopathy NMOs. **a**, List of top ten compounds exhibiting the most inverse correlation with GNE disease gene expression patterns in MCF7 cells (left) and corresponding mechanisms of action (MOA) (right). Compounds classified as Pi3k/mTOR inhibitors are indicated in red. **b**, Dot plot of drugs exhibiting opposing gene expression patterns to GNE disease across nine cell lines, grouped by MOA class. Compounds classified as Pi3k/mTOR inhibitors are indicated in red. **c**, Immunoblot analysis of KO cells showing LC3B(I/II) and phosphorylation of AKT(S473), ULK1(S757: inhibitory) after 8 h of glucose starvation, with or without 1.0 μM copanlisib. **d**, Schematic representation of the establishment of an isogenic NMOs pair derived from normal and I329T hPS cells with bright-field images of representative organoids at day 25. **e**, Immunofluorescence images of normal and I329T NMOs stained with SNA (green), MYOD (red) and DAPI (blue). **f**, Heatmap of gene expression patterns for the ‘AUTOPHAGY VESICLE NUCLEATION ELONGATION MATURATION MTORC1 Pi3kC3’ gene set in normal and I329T NMOs. **g**, Immunofluorescence images of normal and I329T NMOs stained with LC3B (green), Fast MyHC (red) and DAPI (blue) under mock and 4-h EBSS starvation under Bafilomycin A1 (100 nM) conditions. Scale bar, 25 μm. **h**, Immunofluorescence images of I329T NMOs stained with LC3B (green), Fast MyHC (red) and DAPI (blue) under mock, 4-h EBSS + Bafilomycin A1 and 4-h EBSS + Bafilomycin A1 with copanlisib (1.0 μM) conditions day 27 I329T NMOs (left). Graph showing quantification of LC3B-II puncta based on the immunofluorescence images (right). Scale bar, 25 μm. **i**, Immunofluorescence images of normal and I329T NMOs stained with LC3B (green), Fast MyHC (red) and DAPI (blue) under mock and 24-h glucose starvation conditions. Scale bar, 25 μm. **j**, Immunofluorescence images of I329T NMOs stained with LC3B (green), Fast MyHC (red) and DAPI (blue) under mock, 24-h glucose deprivation and 24-h glucose deprivation with copanlisib (1.0 μM) conditions in day 46 I329T NMOs (left). Graph showing quantification of LC3B-II puncta based on the immunofluorescence images (right). Scale bar, 25 μm.

As an alternative strategy, we defined transcriptome signatures representing pathogenicity (that is, a pathogenic gene signature). Using the CMap dataset (https://clue.io/about), we then examined FDA-approved drugs whose transcriptome signatures inversely correlated with the pathogenic signature, as previously described^48,49^. In brief, CMap datasets provide a list of small molecules under preclinical or clinical development for which transcriptome data from treated cells is available; these data are scored on their inverse relation to a ‘pathogenic gene signature’, and the molecules with best-matching profiles are selected^50^. A total of 964 overlapping genes were identified from two datasets: DEGs of mutant compared with normal myoblasts respectively derived from I329T and normal hES cells, and landmark genes from the CMap dataset, which includes drug treatment data across cell lines (Supplementary Fig. 6c). To assess signature similarity, we applied two methods to the of log_2_FC vectors, (1) correlation and (2) cosine similarity, as previously described (Supplementary Fig. 6d). In both methods, a negative value indicated a drug that counteracted the pathogenic signature, whereas a positive value suggested the drug to exhibit effects aligned with the pathogenic signature. Interestingly, this unbiased in silico screening revealed that the highest scores for reversing pathogenic gene signatures in MCF7 (Fig. 7a), A549 (Supplementary Fig. 6e), and PC3 (Supplementary Fig. 6f) cell lines were predominantly achieved by small-molecule inhibitors of either PI3K or mTOR (Fig. 7b). These predictions were consistent with the effect of mTOR inhibition on autophagy in KO cells (Fig. 6g). Among the various PI3K and mTOR inhibitors currently under preclinical or clinical study (Supplementary Fig. 6g), we selected copanlisib, an FDA-approved selective PI3K inhibitor for leukemia treatment, as a feasible drug candidate for patients with GNE myopathy on account of its immediate market availability. As anticipated, treatment of KO cells with copanlisib reduced both AKT active phosphorylation and ULK1 inhibitory phosphorylation, thereby inducing autophagy (Fig. 7c and Supplementary Fig. 7a). In contrast to SA supplementation, copanlisib did not restore global sialylation levels in KO cells (Supplementary Fig. 7b), indicating that autophagy rescue by copanlisib occurs downstream of the primary hyposialylation defect.

To assess the translational therapeutic potential of copanlisib, we generated three-dimensional self-organizing NMOs from both normal and I329T hES cells, following established protocols^28^ (Fig. 7d). The resulting isogenic NMOs (normal versus I329T NMOs) recapitulated key structural features of the neuromuscular system, comprising distinct muscle and neural compartments (Fig. 7d, inserted box). Immunostaining confirmed the presence of well-organized skeletal muscle tissue, marked by expression of myosin skeletal fast MyHC, and of neuronal regions, enriched for TUJ1-positive neurons (Supplementary Fig. 7c). Notably, these neurons extended projections into the adjacent muscle domain, indicating successful innervation of muscle fibers (Supplementary Fig. 7d). In addition, NMOs displayed an enrichment of MYOD-positive myoblasts in the muscle region at day 27 (Supplementary Fig. 7e), with a relatively reduced abundance at day 46 (Supplementary Fig. 7f), in line with reported early myogenic features of pre-day 50 NMOs^28^. Hyposialylation, a hallmark pathophenotype of GNE myopathy, was clearly observed in the muscle region of I329T NMOs (Fig. 7e). Consistent with findings in hES cell-derived myoblasts (Fig. 1e), transcriptomic analysis of normal versus I329T NMOs revealed distinct separation by PCA (Supplementary Fig. 7g), along with enrichment of autophagy-related gene sets (Fig. 7f and Supplementary Fig. 7h). Furthermore, enhanced mTORC1 signaling—previously shown to underlie defective autophagy in the C2C12 KO model (Fig. 6)—was also evident in I329T NMOs, as demonstrated by enrichment of mTORC1-associated gene sets (Supplementary Fig. 7i,j).

Consistently, defective autophagy in I329T NMOs was demonstrated by reduced LC3B punctation, a hallmark of autophagosome formation, following EBSS treatment in the presence of bafilomycin A1 (Fig. 7g), whereas normal NMOs showed robust induction of autophagosome formation under the same conditions (Supplementary Fig. 7k). Given that the I329T NMO model, derived from I329T hESCs, faithfully recapitulates key cellular and molecular hallmarks of GNE myopathy—including hyposialylation, impaired autophagy and elevated mTORC1 signaling—we next assessed whether pharmacological intervention with copanlisib could restore autophagic function and mitigate disease-associated phenotypes. Copanlisib, identified via CMap analysis (Fig. 7) and previously shown to reverse inhibitory ULK1 phosphorylation in the C2C12 KO model (Fig. 7c), significantly rescued autophagosome formation in early-stage I329T NMOs (D27) under EBSS plus bafilomycin A1 treatment (Fig. 7h). A similar rescue effect was observed in more differentiated I329T NMOs at day 46, including MyHC-positive myocytes exhibiting a distinct striated banding pattern, as indicated by restored LC3B punctation compared with untreated I329T NMOs (Figs. 7i,j and Supplementary Fig. 7k).

### Validation of the impaired autophagy using human clinical transcriptomic data

To further validate the clinical relevance of the mechanistic axis identified in our in vitro and organoid models, we analyzed publicly available transcriptomic data from patients with GNE myopathy (GSE12648)^51^. This dataset comprises skeletal muscle biopsies spanning multiple clinical variables, including sex, age, tissue origin and pathological stage (Supplementary Fig. 8a). PCA revealed substantial heterogeneity across samples (Supplementary Fig. 8b); however, disease pathology stage (graded 1–3) emerged as the dominant contributor to variance along PC1, consistent with progressive disease severity. To minimize confounding effects arising from clinical and demographic heterogeneity, we stratified samples based on PC1 extremes, defining a PC1_low group enriched for histologically normal or mildly affected samples and a PC1_high group corresponding predominantly to advanced pathological stages (grades 2–3). Differential expression analysis between PC1_high and PC1_low revealed robust transcriptional changes characteristic of muscle pathology, including alterations in muscle development, contractile apparatus organization and cytoskeletal regulation (Supplementary Fig. 8c,d).

Importantly, GSEA demonstrated that PC1_high samples exhibited significant enrichment of autophagy- and lysosome-related pathways, including vacuolar acidification, lysosomal organization, lytic vacuole organization and macroautophagy (NES >1.7; Supplementary Fig. 8e). In parallel, pathways associated with ECM remodeling and integrin-mediated signaling were also strongly enriched in PC1_high samples (Supplementary Fig. 8f), together with gene sets related to ECM production, most notably collagen fibril organization (NES ~2.06; Supplementary Fig. 8g).

Strikingly, this transcriptional landscape observed in human patient muscle closely mirrors the molecular signatures identified across our three independent disease models—hPS cell-derived myoblasts, *Gne* KO C2C12 myoblasts, and NMOs—where excessive ECM production, integrin/FAK signaling and aberrant PI3K–AKT–mTORC1 activation converged to suppress ULK1-dependent autophagy initiation. Thus, the enrichment of ECM–integrin signaling and autophagy-related pathways in PC1_high patient samples provides independent clinical support for a conserved disease axis linking ECM remodeling to impaired autophagic flux in GNE myopathy.

## Discussion

Generation of isogenic hPS cell pairs and their differentiation into disease-relevant cell types—often referred to as ‘disease-in-a-dish’ systems—has emerged as a powerful strategy for modeling human disease mechanisms^52^. However, large-scale biochemical and mechanistic studies using hPS cell-derived cells remain labor-intensive, underscoring the need for a complementary and tractable model that faithfully recapitulates key disease phenotypes.

Guided by transcriptomic insights from two independent hPC cell-based GNE myopathy datasets^16,17^, we used a *Gne*-KO C2C12 myoblast model. Importantly, the I329T mutation used in this study has been shown to result in near-complete loss of GNE enzymatic activity^17^, effectively functioning as a loss-of-function allele. On this basis, the C2C12-KO model represents an appropriate and mechanistically tractable system to interrogate disease-relevant phenotypes initially identified in I329T hPS cell-derived myoblasts. Consistent with this rationale, impaired autophagy and aberrant AKT–mTORC1 signaling observed in C2C12 KO cells were subsequently recapitulated in I329T NMOs.

Autophagy emerged as a prioritized pathogenic axis based on its consistent enrichment across hPS cell-derived transcriptomes and its established pathological relevance in GNE myopathy, where rimmed vacuoles reflect defective autophagic flux and accumulation of undegraded material. This focus is supported by multiple patient-derived studies reporting abnormal autophagy, protein aggregation and rimmed vacuoles^30–33^. Integrating transcriptomic and biochemical analyses identified excessive ECM production and focal-adhesion signaling as upstream drivers of noncanonical AKT–mTORC1 activation, leading to sustained ULK1 inhibition despite AMPK activation. Given that persistent ULK1 inhibition is a recognized contributor to myopathy^53^, targeting the AKT–mTORC1 axis emerged as a more promising strategy than AMPK activation alone.

Unbiased drug prediction using CMap analysis highlighted PI3K and mTOR inhibitors as compounds capable of reversing the pathogenic gene signature, a prediction that aligned closely with our experimental findings. Considering the known risk of muscle atrophy associated with direct mTOR inhibition^54^, we focused on PI3K inhibition and selected copanlisib, an FDA-approved PI3K inhibitor with favorable pharmacokinetics and a manageable safety profile^55–57^. Copanlisib effectively suppressed AKT–mTORC1 signaling and restored ULK1-dependent autophagy in C2C12 cells, and importantly, these effects translated to three-dimensional NMOs derived from I329T hES cells, where copanlisib rescued defective autophagosome formation.

NMOs provide a critical bridge between simplified two-dimensional systems and in vivo models by enabling tissue-level validation of disease mechanisms and therapeutic responses. Although overt histopathological hallmarks such as rimmed vacuoles were not observed—consistent with the developmental immaturity of NMOs, which transcriptionally correspond to approximately 17-week human fetal skeletal muscle^58^—this system robustly recapitulated early, muscle-intrinsic pathogenic processes, including impaired autophagy under metabolic stress and aberrant AKT–mTORC1 signaling.

In summary, this study establishes an integrated disease-modeling pipeline in which hPS cell-based systems enable discovery, a C2C12-KO model supports mechanistic dissection, and NMOs provide tissue-level validation of therapeutic efficacy. Together, this framework identifies defective autophagy driven by noncanonical AKT–mTORC1 activation as a druggable vulnerability in GNE myopathy and highlights copanlisib as a promising proof-of-concept therapeutic candidate.

## Supplementary information

Supplementary Information

## Data Availability

Source data are available from the corresponding authors upon request. The bulk RNA-seq data are available from NCBI Gene Expression Omnibus under accession number GSE278862.
