# Supplementary material for: Defective autophagy in GNE myopathy is rescued by inhibition of noncanonical Akt–mTORC1 activation across multiple isogenic models
Source: Exp Mol Med. 2026 Apr 10;58(4):1187–202. doi: 10.1038/s12276-026-01701-7 (PMC13144418; doi:10.1038/s12276-026-01701-7)
Supplement: Supplementary file 1 — Supplementary Information [file 12276_2026_1701_MOESM1_ESM.pdf]

Defective Autophagy in GNE Myopathy is Rescued by Inhibition of Noncanonical Akt–mTORC1 Activation across Multiple Isogenic Models

Running title: Disease-in-a-Dish Modeling of GNE Myopathy

Dong-Woo Kim, Eun-Ji Kwon, Hyuk Kwon, Jumee Kim, and Hyuk-Jin Cha

Supplementary Fig. 1

a

Dataset	Cell lines	Sample Size	Samples	Samples	PMID
In-house	H9 Human embryonic stem cells (hESC)	10	Normal myoblasts (2)<br>Mutant myoblasts (8)<br>from hESCs	Normal, I329T, R160Q, I588T, V727M	35202935
PRJNA 739168	Human iPSCs from GNE myopathy patient and healthy control	9	Normal myoblasts (6)<br>Mutant myoblasts (3)<br>from iPSCs	Control 1, Control 2, GNE	36085325

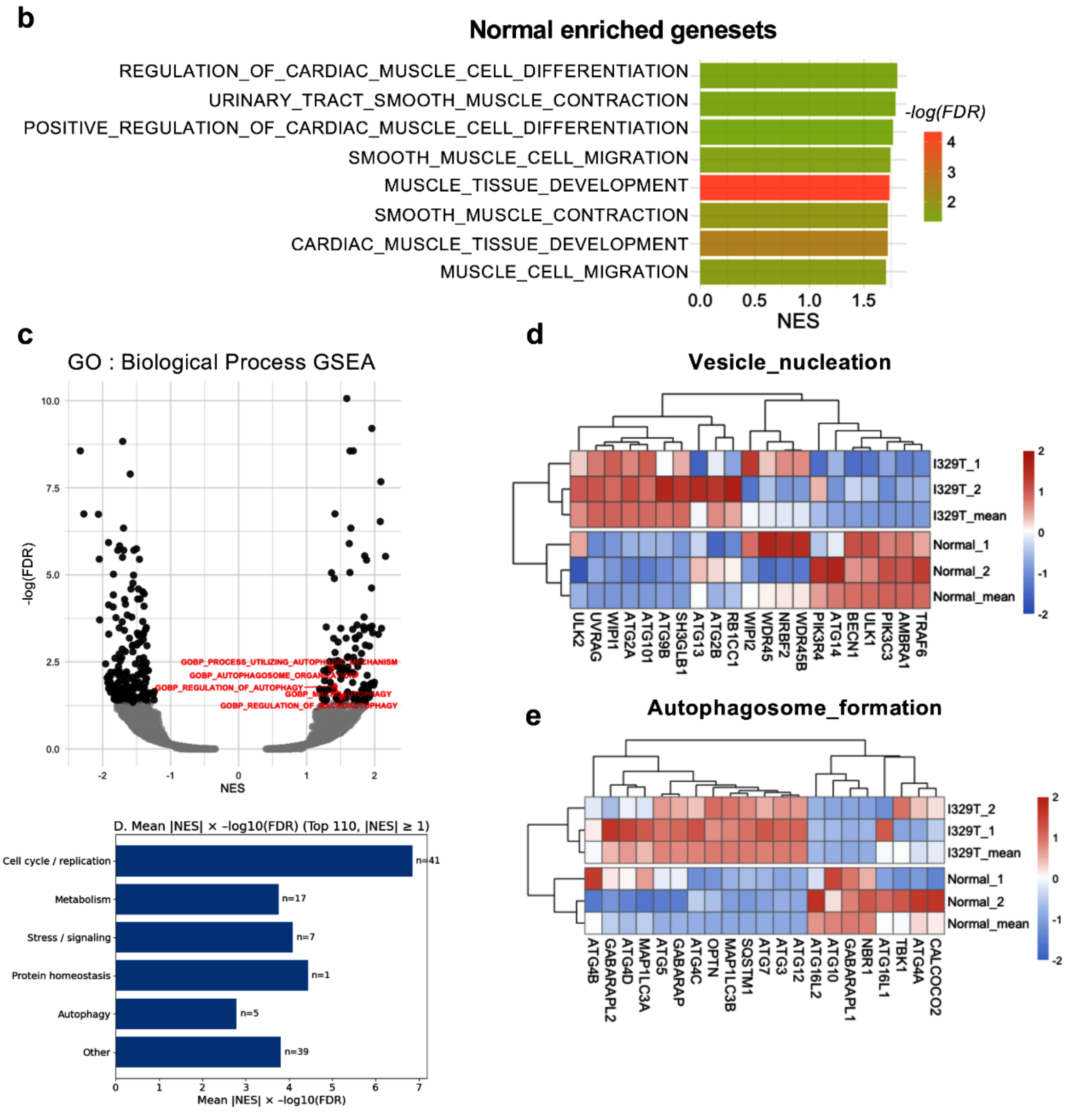

Supplementary Fig. 2

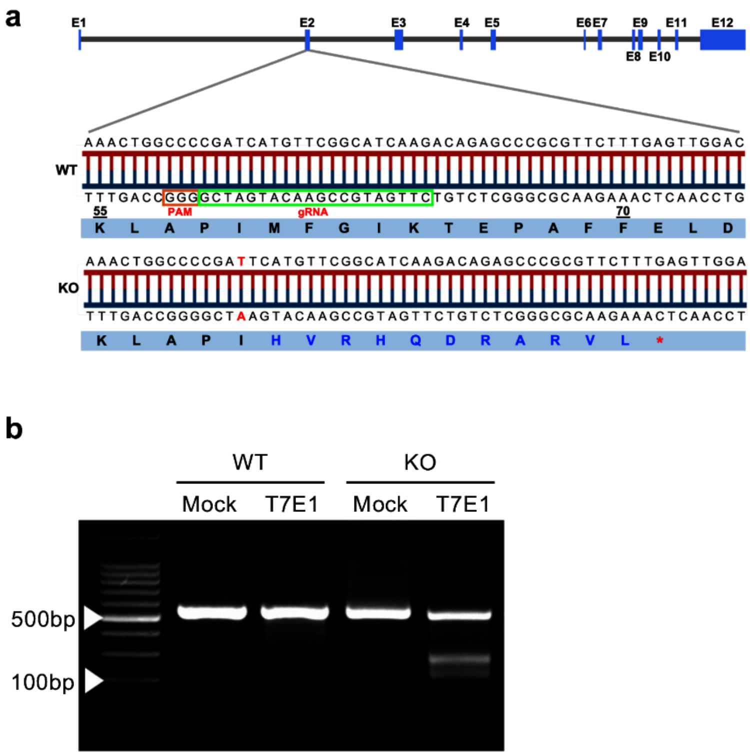

Supplementary Fig. 3

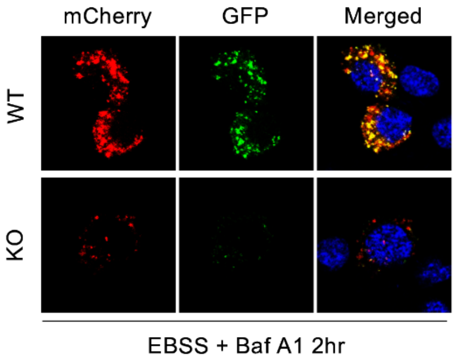

Supplementary Fig. 4

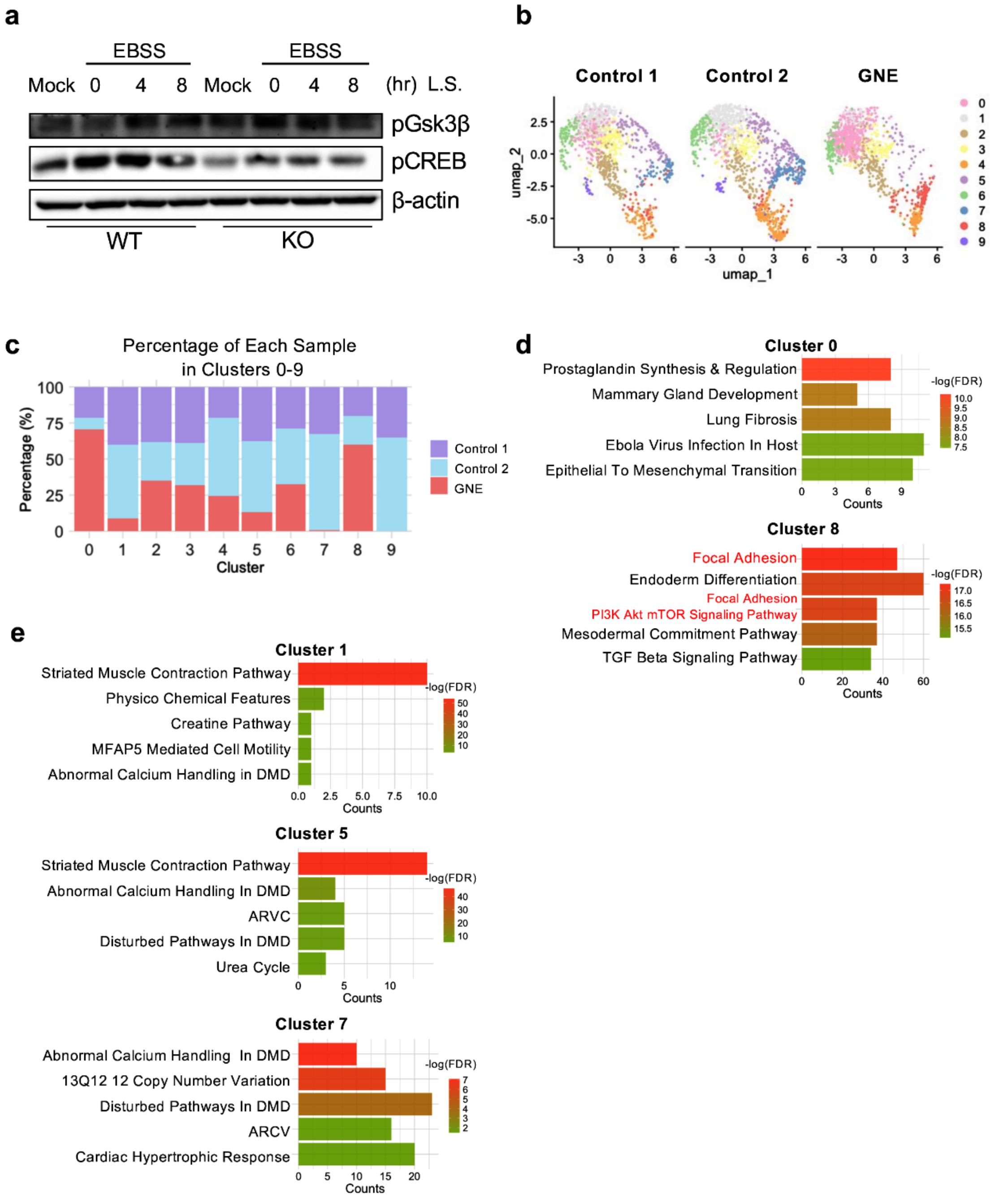

Supplementary Fig. 5

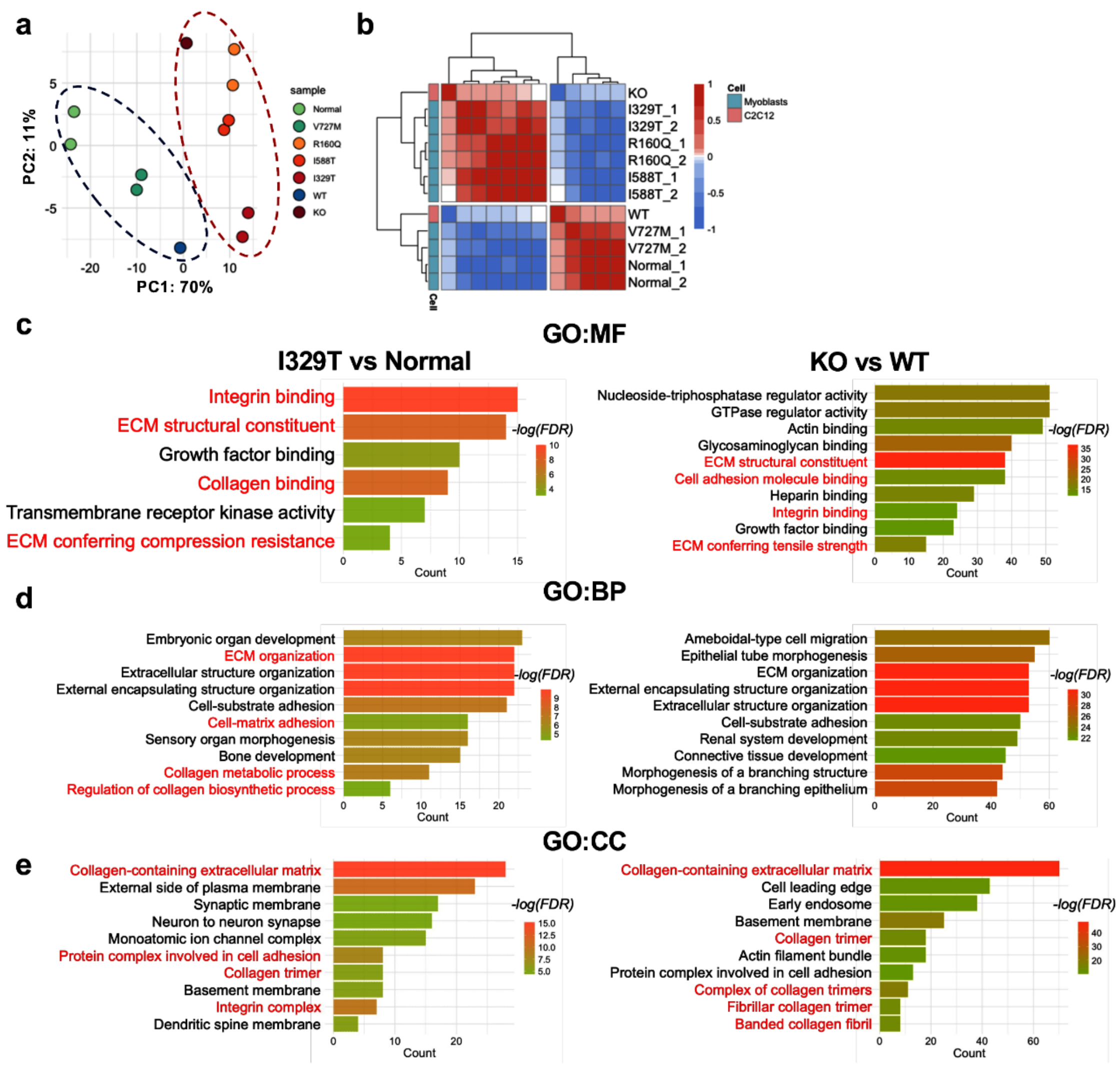

Supplementary Fig. 6

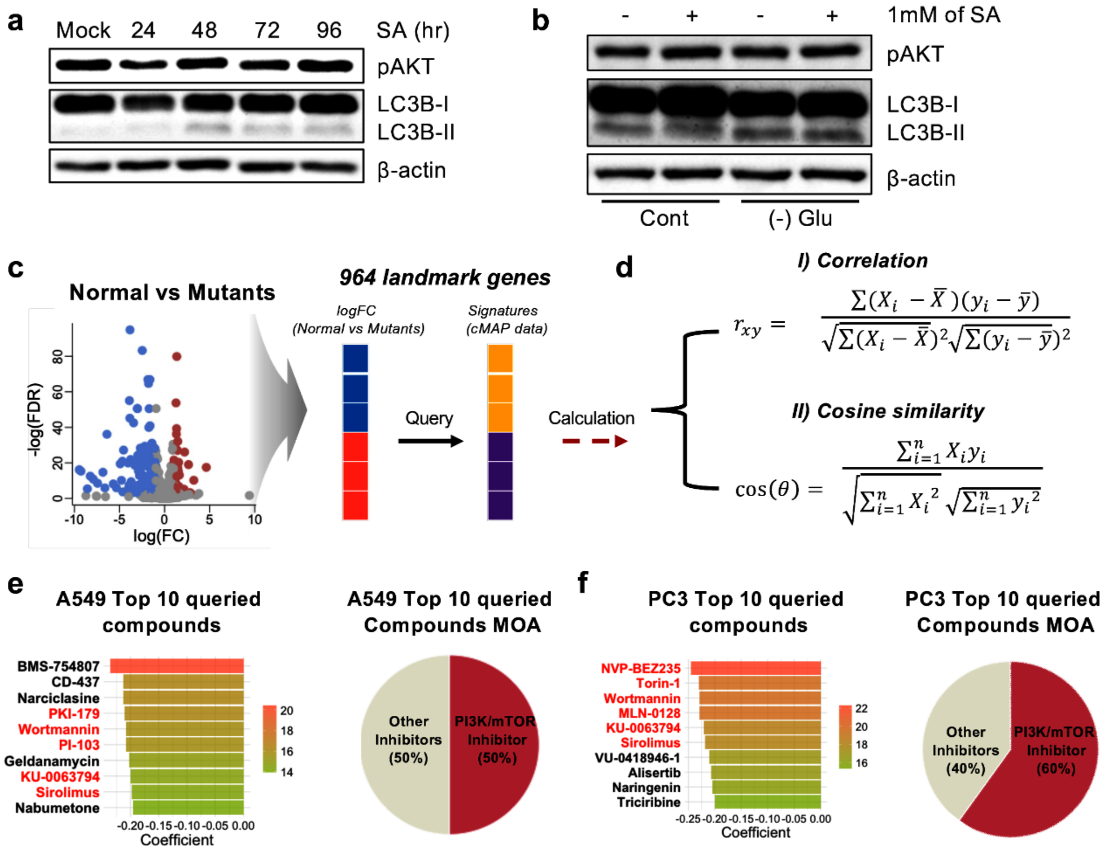

g

		Compound	Drug	Target	IC50	Application
FDA-approved		BAY80-6946	Copanlisib	PI3K	0.5nM	Leukemia
FDA-approved		Sirolimus	Rapamycin	mTOR	0.1nM	Immunosuppressant
Clinical	Ph. 1	MLN-0128	Sapanisertib	mTOR	1nM	
	Ph. 2	GDC-0980	Apitolisib	PI3K<br>mTOR	5nM<br>17nM	
	Ph. 1/2	SAR245409	Voxtalisib	mTOR/PI3K	9nM	
	Ph. 1/2	AZD2014	Vistusertib	mTOR1/2<br>PI3K	2.5nM<br>3.8μM	
	Ph. 1	GSK2126458	Omipalisib	PI3K<br>mTOR1/2	0.019nM<br>0.18/0.3nM	
	Ph. 2	NVP-BEZ235	Dactolisib	PI3K<br>mTOR	4nM<br>6nM	Autophagy, HIV
Pre-clinical		Torin1		mTOR1/2	2/10nM	
		Wortmannin		PI3K<br>DNA-PK/ATM	3nM<br>16/150nM	
		KU-0063794		mTOR1/2	10nM	
		AS-0605240		PI3K	8nM	
		PKI-179		PI3K<br>mTOR	8nM<br>0.42nM	Anti-tumor
		PI-103		PI3K<br>mTOR/DNA-PK	2nM<br>30/23nM	Apoptosis

Supplementary Fig. 7

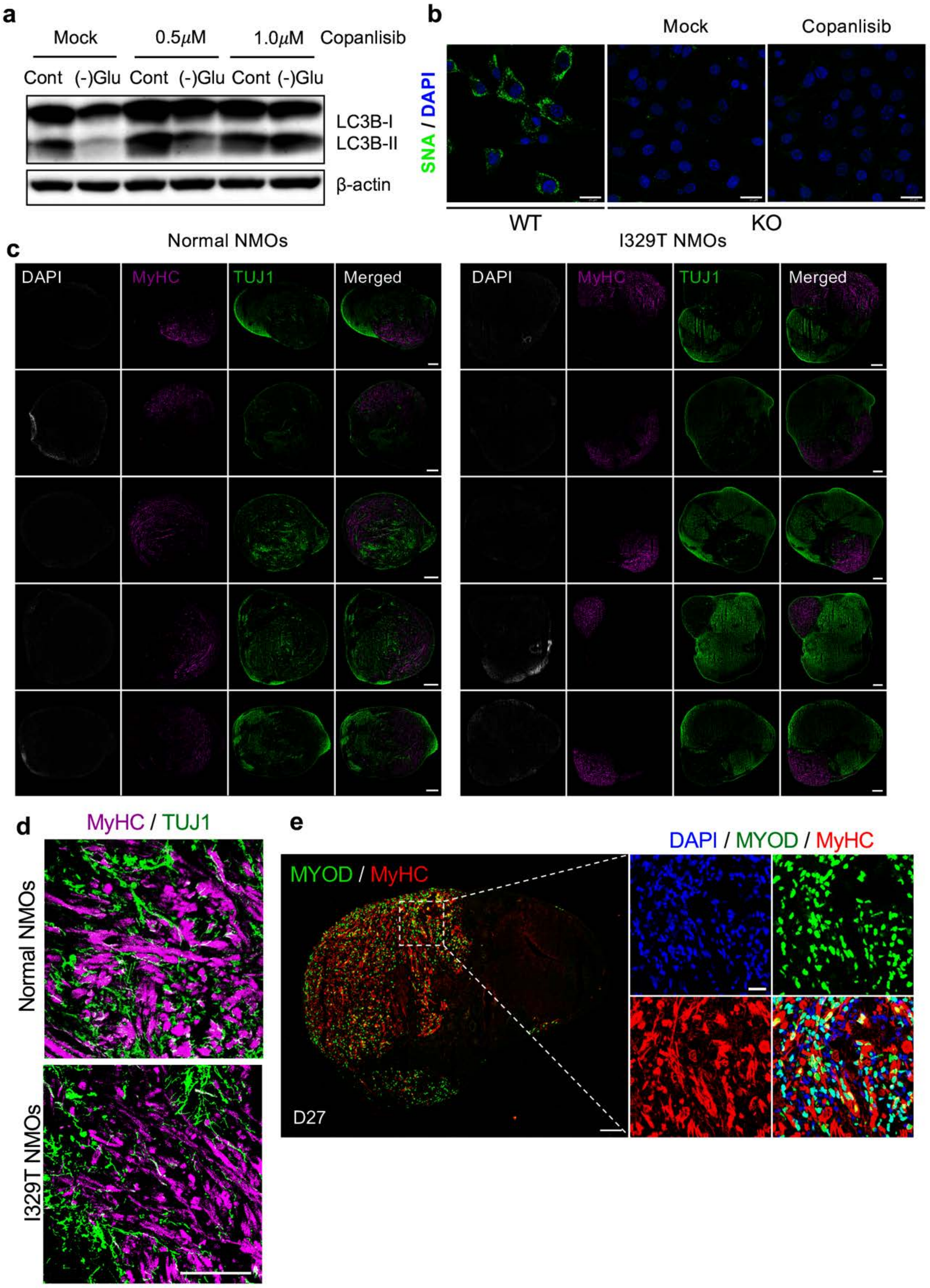

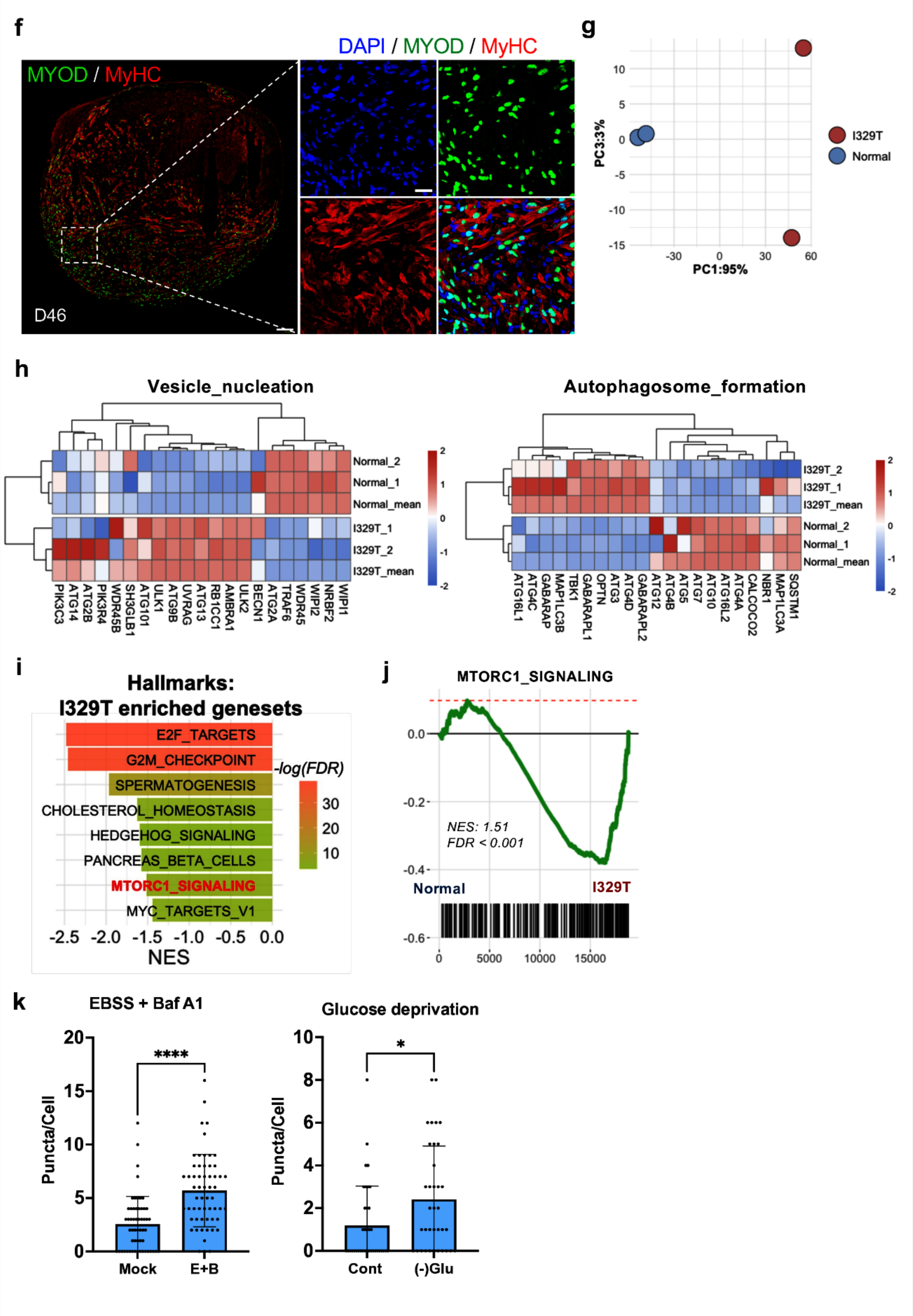
f
MYOD / MyHC
D46
DAPI / MYOD / MyHC
g
PC3:3%
PC1:95%
I329T
Normal
h
Vesicle_nucleation
Autophagosome_formation
Normal_2
Normal_1
Normal_mean
I329T_1
I329T_2
I329T_mean
PIK3C3
ATG14
ATG2B
PIK3R4
WDR45B
SH3GLB1
ATG101
ULK1
ATG9B
UVRAG
ATG13
RB1CC1
AMBRA1
ULK2
BECN1
ATG2A
TRAF6
WDR45
WIPI2
NRBF2
WIPI1
ATG16L1
ATG4C
GABARAP
MAP1LC3B
TBK1
GABARAPL1
OPTN
ATG3
ATG4D
GABARAPL2
ATG12
ATG4B
ATG5
ATG7
ATG10
ATG16L2
ATG4A
CALCOCO2
NBR1
MAP1LC3A
SQSTM1
i
Hallmarks:
I329T enriched genesets
E2F_TARGETS
G2M_CHECKPOINT
SPERMATOGENESIS
CHOLESTEROL_HOMEOSTASIS
HEDGEHOG_SIGNALING
PANCREAS_BETA_CELLS
MTORC1_SIGNALING
MYC_TARGETS_V1
-log(FDR)
NES
j
MTORC1_SIGNALING
NES: 1.51
FDR < 0.001
Normal
I329T
k
EBSS + Baf A1
Glucose deprivation
Puncta/Cell
****
*
Mock
E+B
Cont
(-)Glu

Supplementary Fig. 8

a

#	Clinical status	Pathology stage	Tissue type	Age	Gender
317365		2	Deltoid	26	M
317366		3	Deltoid	55	F
317367		2	Deltoid	45	M
317368		1	Deltoid	29	M
317369		1	Biceps	29	M
317370	A	2	Quadriceps	59	F
317371		2	Deltoid	40	M
317372		2	Deltoid	46	F
317373		2	Biceps	25	F
317374		3	Tibialis anterior	26	M
317375		1	Quadriceps	18	M
317376		1	Quadriceps	52	F
317377		1	Gluteus	56	M
317378		1	Deltoid	26	M
317379		1	Deltoid	19	M
317380	N	1	Paraspinal	73	F
317381		1	Triceps	31	F
317382		1	Deltoid	74	F
317383		1	Quadriceps	21	M
317384		1	Triceps	48	F

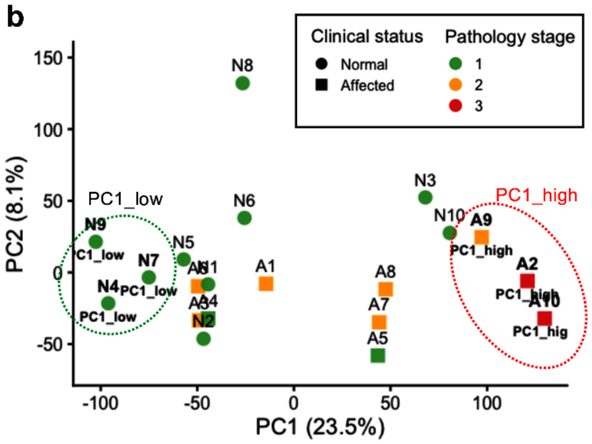

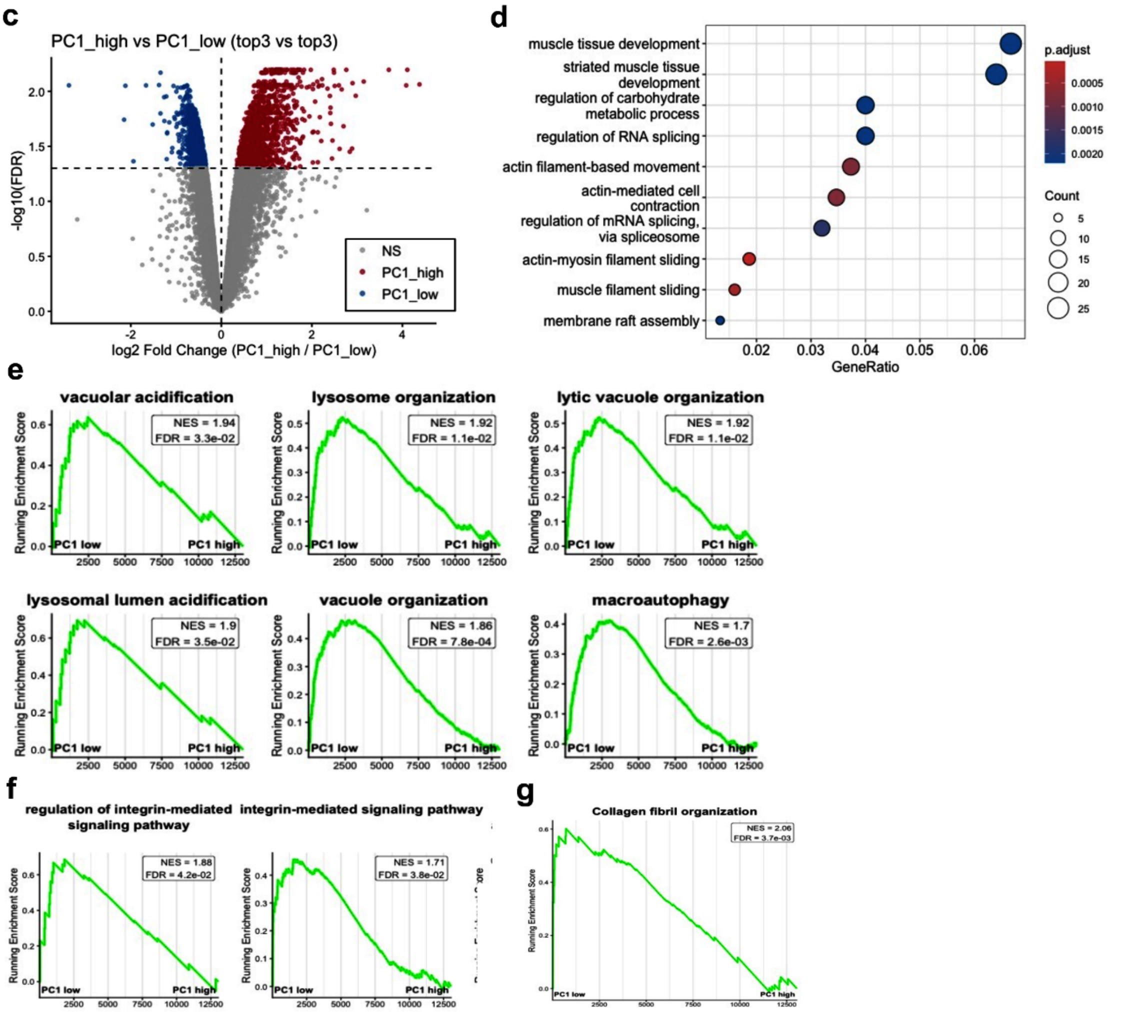

Supplementary Materials and Methods

Bulk RNA-seq library preparation

Total RNA was isolated from C2C12 cells using Easy-BLUE^{TM} RNA isolation kit (iNtRON Biotechnology, #17061). 1 μg of total RNA was processed for preparing mRNA sequencing library using MGIEasy RNA Directional Library Prep Kit (MGI) according to manufacturer's instruction. The first step entails utilizing poly-T oligo-attached magnetic beads to isolate the mRNA molecules that contain poly-A. Following purification, divalent cations and a high temperature are used to break the mRNA into small pieces. Utilizing reverse transcriptase and random primers, the cleaved RNA fragments are converted into first strand cDNA. After achieving strand specificity in the RT directional buffer, second strand cDNA synthesis takes place. The 'A' base is then added to these cDNA fragments, followed by the ligation of the adapter. The final cDNA library is made by purifying and enriching the results with PCR. The QauntiFluor ONE dsDNA System (Promega) is used to quantify the double stranded library. The library is circularized at 37 °C for 30 min, and then digested at 37 °C for 30 min, followed by cleanup of circularization product. The library is treated with the DNB enzyme at 30 °C for 25 min to create DNA nanoballs (DNB). Finally, Library was quantified by QauntiFluor ssDNA System (Promega). On the MGIseq system (MGI), the prepared DNB was sequenced using 100 bp paired-end reads.

Organoid samples were dissociated into single cells prior to RNA extraction. Briefly, organoids were incubated with 1 mL of Liberase™ TL Research Grade (Roche) at 37 °C for 30 min according to the manufacturer's protocol. After enzymatic digestion, organoids were mechanically dissociated by repeated pipetting until a single-cell suspension was obtained, and the suspension was passed through a 40 μm cell strainer (Falcon®) to remove aggregates and debris. Total RNA was then isolated from the resulting cell suspension using the Easy-BLUETM RNA isolation kit (iNtRON Biotechnology, #17061). From this Easy-BLUETM

RNA isolation step onward (including mRNA library preparation, quantification, circularization/DNB generation, and MGIseq sequencing), the procedure was identical to that described above for C2C12 cells.

Transfection for establishment of GNE Knock-Out myoblasts

For transfection, C2C12 myoblasts were rinsed with DPBS and detached using 0.25% trypsin-EDTA. Cells were washed three times with Opti-MEM (31985070, Gibco). The cells were then counted, and 1×10^6 cells were resuspended in 100 µL of Opti-MEM. 2 µg of spCas9 plasmid and 3 µg of sgRNA plasmid vectors were added to the cell suspension. The cell and plasmid mixture were transferred to an electroporation cuvette. Electroporation was performed using a Nepa-21 electroporator with a poring pulse of 200 V and a transfer pulse of 2.5 ms. The transfected cells were seeded into a 6-well plate filled with DMEM culture medium. For single-cell isolation, the cells were diluted to a concentration of 1 cell per µL in culture medium, and 10 µL of this cell solution was seeded into 100 pi culture dishes. Single clones were isolated and expanded first in 12-well plates and then in 6-well plates. The Gne KO C2C12 myoblast clones were analyzed by Sanger sequencing.

Flow cytometry

Cells were detached with 0.25% trypsin-EDTA for C2C12 followed by three washes with DPBS and then analyzed using FACS Calibur, Celesta, and Fortessa (BD Biosciences). For the determination of sialic acid, cells were stained with 1:400 fluorescein-labeled SNA (FL-1301, Vector Laboratories) and 1:330 biotinylated MALII (B-1265, Vector Laboratories). For detection of glycospingolipids, cells were stained with 1:1000 BODIPY 493/503 (2 µM, Invitrogen). CellQuest Pro software and FlowJo software were used for FACS analysis.

Immunocytochemistry

Cells were treated with cold methanol or 4% PFA/0.5% Triton X-100 for fixation and permeabilization. 3% BSA in PBS was used for blocking solution. For lectin staining,

fluorescein-labeled SNA (FL-1301, Vector Laboratories) was diluted in blocking solution and applied to cells for 1 h at room temperature in the dark. In the case of MALII staining, cells were washed three times and then incubated with 1:200 Alexa Fluor 594 streptavidin (#405240, BioLegend) for 30 min at room temperature in the dark. Cells were washed four times before nuclei were stained with DAPI (Thermo Fisher) and mounted on slide glass using MOWIOL solution. Confocal Scope TCS SP8 (Leica) was used for imaging samples.

Immunoblotting analysis

Cells were detached with 0.25% trypsin -EDTA or cell scraper (#3008, Corning) followed by three times DPBS wash on ice. RIPA buffer (Biosesang) containing 1 μM protease inhibitor and 10 μM sodium orthovanadate was used to extract the whole cell lysate, which was then collected after incubating on ice for 1 hour followed by centrifugation. Protein concentration was measured using the Pierce BCA protein assay Kit (#23225, Thermo Fisher Scientific). Protein lysates were diluted with 5× SDS-PAGE loading buffer (SF2088-110-00, Biosesang) and RIPA buffer. Then, protein samples were boiled at 100 °C for 10 minutes. 10-20 μg of prepared protein sample was loaded and separated on a 7.5-10% SDS-PAGE gel. The separated proteins were transferred to an activated PVDF membrane. 5% skim milk in TBS-T was used for the blocking solution. The transferred membranes were blocked with blocking solution at RT for 1 hour followed by three times TBS-T wash then incubated with primary antibody (1:200-1:1000) in TBS-T at 4 °C overnight. Then, the membranes were washed three times with TBS-T and incubated with secondary antibody (1:10000) in TBS-T at room temperature for 1 hour. Chemiluminescence was detected using Miracle-Star (#16028, iNtRON Biotechnology) or Amersham ECL Prime (#GERPON2232, Cytiva).

RT-PCR analysis

Easy-BLUE™ RNA isolation kit (iNtRON Biotechnology) was used for total RNA

extraction. For reverse transcription, 5× PrimeScript™ RT mix (TaKaRa) was used to generate cDNA. Quantitative real-time PCR was performed with SYBR Green PCR reagents (Life Technologies) using the QuantStudio3 (Applied Biosystems).

Cell growth analysis

Cell growth was monitored using the JuLI™ Stage (NanoEntek), an automated real-time live-cell imaging system. Cells were seeded in 6-well plate and cultured in DMEM supplemented with 10% FBS and 50 μg/ml Gentamicin in 5% CO2 at 37°C. In the case of glucose deprivation, cells were washed three times with DPBS and changed to DMEM, no glucose (#11966025, Gibco) with 10% FBS and 50 μg/ml gentamicin. The JuLI™ Stage was placed inside the incubator, and time-lapse imaging was performed at 1-4 hour intervals in bright field mode.

Collagen quantification assay

Total soluble collagen content was measured using the Sircol™ Soluble Collagen Assay Kit (Biocolor, UK) according to the manufacturer's instructions. Cell culture supernatants were collected 3-4 days after seeding. The Multi Mixer (SLRM-3, SeouLin Bioscience) was used for inverting steps during the collagen assay. Collagen concentrations were measured using the Epoch Microplate Spectrophotometer (Biotek).

Acquisition of Public Clinical Transcriptome Data

Publicly available microarray data were retrieved from the Gene Expression Omnibus (GEO) database under accession number GSE12648. This dataset comprises raw intensity profiles from skeletal muscle biopsies (deltoid, biceps, quadriceps, and tibialis) of 10 patients with Hereditary Inclusion Body Myopathy (HIBM) and 10 normal controls. The HIBM patients harbored the homozygous M712T GNE mutation (the Persian Jewish founder allele).

Supplementary Figure Legends

Supplementary Fig. 1 (a) Summary of GEO datasets used for cross-validation, including sample types, sizes, and relevant PMIDs. **(b)** Gene ontology terms enriched in Normal group compared to I329T, highlighting muscle development and smooth muscle contraction pathways. **(c)** Volcano plot displaying GSEA results (GO:BP) for genes regulated in a severity-dependent manner (Normal to I329T). Autophagy-related gene sets are marked in red. The gradient of severity was defined as Normal < V727M < R160Q/I588T < I329T. GO BP terms (top 110 by FDR, |NES| ≥ 1) were categorized and quantified using an integrated enrichment score (mean |NES| × –log10(FDR)) between Normal vs I329T. Autophagy-related pathways show consistent enrichment strength despite smaller pathway counts, supporting focused mechanistic interrogation. **(d, e)** Enrichment of gene sets related to vesicle nucleation (d) and autophagosome formation (e) in Normal versus I329T sample.

Supplementary Fig. 2 (a) Graphical description of the gRNA genomic locus, along with the DNA and amino acid sequences of both WT and KO cells. The T-A base insertion is shown in red. Altered amino acid sequences are indicated in blue. **(b)** Schematic of T7 endonuclease I (T7E1) digestion assay showing genome editing in WT and KO cells under mock and T7E1 conditions. Gel electrophoresis of PCR-amplified genomic DNA from WT and KO cells, confirming indel formation.

Supplementary Fig. 3 Representative images of mCherry-GFP-LC3 fluorescence in WT and KO cells following 2-hour EBSS and Bafilomycin A1 treatment. Yellow puncta indicate autophagosomes, and red-only puncta represent autolysosomes.

Supplementary Fig. 4 (a) Immunoblot analysis of WT and KO cells showing Gsk3β phosphorylation and CREB phosphorylation under mock conditions and after 0, 4, and 8 hours

of EBSS treatment. Mock: DMEM with 10% FBS; L.S.: EBSS with 1% FBS. **(b)** Two-dimensional uniform manifold approximation and projection (UMAP) visualization of scRNA-seq data from myoblasts differentiated from GNE myopathy patient iPSCs and healthy controls iPSCs. Each point represents a cell, with colors based on subtype annotations defined by the Louvain clustering algorithm and marker genes. **(c)** Proportion of data types shown in (A) across different clusters. **(d, e)** Enriched MSigDB WIKIPATHWAYS gene sets for upregulated genes in each cluster.

Supplementary Fig. 5 (a) PCA analysis of Myoblasts samples including Normal, V727M, R160Q, I588T, I329T, and C2C12 WT and KO samples. **(b)** Heatmap showing unsupervised hierarchical clustering of pairwise Pearson correlations between the RNA-seq datasets used in (A). **(c)** Enriched Gene Ontology Molecular Function gene sets for upregulated genes in I329T cells compared to Normal cells (left) and KO cells compared to WT cells (right). **(d)** Enriched Gene Ontology Biological Process gene sets for upregulated genes in I329T cells compared to Normal cells (left) and KO cells compared to WT cells (right). **(e)** Enriched Gene Ontology Cellular Component gene sets for upregulated genes in I329T cells compared to Normal cells (left) and KO cells compared to WT cells (right).

Supplementary Fig. 6 (a) Immunoblot analysis of KO cells showing AKT (S473) phosphorylation and LC3B (I/II) levels following 1 mM sialic acid (SA) treatment for the indicated times. **(b)** Immunoblot analysis of KO cells showing phosphorylation of AKT(S473) and LC3B(I/II) after 24 hours of glucose starvation, with or without 1 mM sialic acid (SA) treatment. **(c)** Volcano plot of differential gene expression between normal and mutant samples used for CMap query generation. Genes were filtered to 964 landmark genes and input into the CMap pipeline. **(d)** Schematic overview of the similarity scoring methodology in CMap, showing both Pearson correlation and cosine similarity equations used to compare gene

signatures. **(e)** Bar plot (left) of the top 10 negatively correlated compounds in the A549 cell line, ranked by enrichment coefficient. PI3K/mTOR inhibitors are highlighted in red. Pie chart (right) summarizes the mechanisms of action (MOA) for these top hits, with 50% classified as PI3K/mTOR inhibitors. **(f)** Bar plot (left) of the top 10 negatively correlated compounds in the PC3 cell line, with 60% of the compounds classified as PI3K/mTOR inhibitors (pie chart, right). **(g)** List of PI3K/mTOR-targeting compounds with drug targets, IC50 values, and clinical application statuses.

Supplementary Fig. 7 (a) Immunoblot analysis of KO cells showing phosphorylation of AKT(S473) after 24 hours of glucose starvation, with 0.5 μM or 1.0 μM copanlisib treatment. **(b)** Immunofluorescence images of WT and Gne KO cells with 8 hours 1.0 μM copanlisib treatment stained with SNA (green) and DAPI (blue). Scale bars, 25 μm. **(c)** Immunofluorescence images of neuromuscular organoids (NMOs) at Day 46 derived from Normal and I329T hPSCs, stained with DAPI (gray), MyHC (magenta), and TUJ1 (green). Scale bars, 200 μm. **(d)** High-magnification images showing reduced MyHC/TUJ1 signal intensity and structural organization in Day 46 I329T NMOs compared to Normal NMOs. Scale bars, 100 μm. **(e, f)** Immunofluorescence images of Normal NMOs at Day 27 (e) and Day 46 (f), stained with DAPI (blue), MYOD (green), and MyHC (red). Scale bars, 100 μm (overview) and 25 μm (inset). **(g)** Principal component analysis (PCA) plot illustrating transcriptomic separation between Normal and I329T NMOs. **(h)** Heatmaps showing gene expression of vesicle nucleation (left) and autophagosome formation (right)–related genes in Normal and I329T NMOs. **(i)** Bar graph of enriched MSigDB hallmark gene sets in I329T NMOs versus Normal, highlighting upregulation of MTORC1_SIGNALING. **(j)** GSEA plot showing significant enrichment of the “MTORC1_SIGNALING” gene set in I329T NMOs (NES = 1.51, FDR < 0.001). **(k)** Quantification of LC3B-II puncta in Day 27 Normal NMOs under 4-hour

EBSS plus Bafilomycin A1 condition (left) and Day 46 Normal NMOs under 24-hour glucose deprivation (right), based on the immunofluorescence images in Figures 7g and i.

Supplementary Fig. 8 (a) Table showing clinical information of individuals classified as healthy controls or patients with GNE myopathy. **(b)** Principal component analysis (PCA) of publicly available transcriptomic data from skeletal muscle biopsies of GNE myopathy patients (GSE12648). Samples are colored by pathology stage (1–3) and shaped by clinical status (normal vs affected). PC1 separates samples largely according to pathology stage, with PC1_low corresponding predominantly to normal samples and PC1_high to affected samples (stages 2–3). **(c)** Volcano plot showing differentially expressed genes between PC1_high and PC1_low groups (top 3 samples per group). Genes upregulated in PC1_high and PC1_low are highlighted in red and blue, respectively, based on an adjusted P value < 0.05. **(d)** Gene Ontology (GO) enrichment analysis of differentially expressed genes between PC1_high and PC1_low samples, highlighting pathways associated with muscle development, cytoskeletal organization, RNA processing, and membrane-associated processes. **(e)** Gene set enrichment analysis (GSEA) demonstrating significant enrichment of autophagy- and lysosome-related pathways in PC1_high samples, including vacuolar acidification, lysosome organization, lytic vacuole organization, lysosomal lumen acidification, vacuole organization, and macroautophagy. Normalized enrichment scores (NES) and false discovery rates (FDR) are indicated within each panel. **(f)** GSEA of integrin-mediated signaling pathways showing enrichment toward the PC1_high end, indicating altered ECM–cell adhesion signaling in affected patient samples. **(g)** GSEA of collagen fibril organization, revealing strong enrichment in PC1_high samples (NES = 2.06, FDR = 3.7×10^{-3}), consistent with enhanced extracellular matrix production and remodeling in GNE patient muscle tissue.
